# Exosomes Derived from M2 Microglial Cells Modulated by 1070‐nm Light Improve Cognition in an Alzheimer's Disease Mouse Model

**DOI:** 10.1002/advs.202304025

**Published:** 2023-09-13

**Authors:** Chengwei Chen, Yuting Bao, Lu Xing, Chengyong Jiang, Yu Guo, Shuangmei Tong, Jiayi Zhang, Liang Chen, Ying Mao

**Affiliations:** ^1^ Department of Neurosurgery, Huashan Hospital, Shanghai Medical College Fudan University Shanghai 200040 China; ^2^ National Center for Neurological Disorders Shanghai 200040 China; ^3^ Shanghai Key Laboratory of Brain Function Restoration and Neural Regeneration Shanghai 200040 China; ^4^ Neurosurgical Institute of Fudan University Shanghai 200040 China; ^5^ Shanghai Clinical Medical Center of Neurosurgery Shanghai 200040 China; ^6^ State Key Laboratory of Medical Neurobiology, MOE Frontiers Center for Brain Science and Institutes of Brain Science Fudan University Shanghai 200032 China; ^7^ Department of Pharmacy, Huashan Hospital, Shanghai Medical College Fudan University Shanghai 200040 China

**Keywords:** alzheimer's disease, exosome, near‐infrared, neuroinflammation, photobiomodulation

## Abstract

Near‐infrared photobiomodulation has been identified as a potential strategy for Alzheimer's disease (AD). However, the mechanisms underlying this therapeutic effect remain poorly characterize. Herein, it is illustrate that 1070‐nm light induces the morphological alteration of microglia from an M1 to M2 phenotype that secretes exosomes, which alleviates the β‐amyloid burden to improve cognitive function by ameliorating neuroinflammation and promoting neuronal dendritic spine plasticity. The results show that 4 J cm^−2^ 1070‐nm light at a 10‐Hz frequency prompts microglia with an M1 inflammatory type to switch to an M2 anti‐inflammatory type. This induces secretion of M2 microglial‐derived exosomes containing miR‐7670‐3p, which targets activating transcription factor 6 (ATF6) during endoplasmic reticulum (ER) stress. Moreover, it is found that miR‐7670‐3p reduces ATF6 expression to further ameliorate ER stress, thus attenuating the inflammatory response and protecting dendritic spine integrity of neurons in the cortex and hippocampus of 5xFAD mice, ultimately leading to improvements in cognitive function. This study highlights the critical role of exosomes derive from 1070‐nm light‐modulated microglia in treating AD mice, which may provide a theoretical basis for the treatment of AD with the use of near‐infrared photobiomodulation.

## Graphical abstract

In this study, 4 J cm^−2^ 1070‐nm light at 10 Hz is used to modulate the shift of microglia from M1 to M2 phenotype which secret exosomes that ameliorate neuroinflammation and promote neuronal dendritic spine plasticity, resulting in improvement of cognition in Alzheimer's disease mouse model.

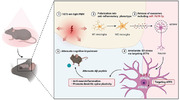

## 1 Introduction

Alzheimer's disease (AD), a type of dementia associated with aging, causes patients to exhibit progressive declines in cognitive abilities, including memory and learning. AD is characterized by synaptic loss, neurodegeneration, and cognitive impairment.^[^ ^1^, ^2^ ^]^ Along with neurofibrillary tangles, the aggregation of β‐amyloid (Aβ) has been found to trigger neuroinflammation and oxidative stress, key factors in AD pathogenesis, resulting ultimately in synaptic degeneration and neuronal loss.^[^ ^2^, ^3^, ^4^ ^]^

Activation of microglia by Aβ can induce chronic inflammation, which in turn exacerbates neurodegenerative pathology through an inflammatory response to Aβ deposits.^[^ ^5^ ^]^ In previous studies, the suppression of neuroinflammation has been shown to improve microglial and lymphatic clearance of Aβ, protect against neuronal loss, and increase synaptic plasticity and cognitive function.^[^ ^6^, ^7^ ^]^ Therefore, effective inhibition of neuroinflammation is one of the key strategies for treating AD, although in vivo anti‐neuroinflammatory treatments are lacking.

Photobiomodulation (PBM) is a form of light therapy that uses visible or near‐infrared (NIR) light with wavelengths that activate cellular processes. Previous studies have demonstrated the efficacy of PBM in treating ischemic stroke, pain, and depression in both animal models and clinical settings.^[^ ^8^, ^9^, ^10^, ^11^ ^]^ Recently, several studies have provided evidence for PBM as a therapeutic strategy to control the progression of AD.^[^ ^12^, ^13^, ^14^ ^]^ By treating mice with a non‐invasive flickering light regimen, Iaccarino et al. observed reduced Aβ levels in the visual cortex of 5xFAD mice.^[^ ^12^ ^]^ Zhang et al. found that Aβ production and plaque formation were reduced by shifting APP processing toward a non‐amyloidogenic pathway in APPswe/PS1dE9 (APP/PS1) mice after 632.8‐nm light therapy, thereby improving memory and cognition.^[^ ^13^ ^]^ Grillo et al. demonstrated that 1072‐nm light therapy reduced Aβ protein levels in the brains of AD mouse models.^[^ ^14^ ^]^ Despite PBM's beneficial effects, its mechanisms remain largely unknown.

The NIR spectral region offers greater tissue penetration, reduced scatter and absorption, and minimal autofluorescence compared to visible light, making it ideal for clinical applications.^[^ ^15^ ^]^ A previous study showed that 1070‐nm light decreased M1‐like microglia and attenuated the Aβ burden in the cortex, thus reversing cognitive impairment in APP/PS1 mice.^[^ ^16^ ^]^ Microglia, the brain's resident immune cells, play a pivotal role in the neuroinflammatory response in AD. Activated microglia can be classified into two phenotypes: M1 inflammatory microglia and M2 anti‐inflammatory microglia; the M1 phenotype can shift to the M2 phenotype under certain conditions, contributing to neuroprotection.^[^ ^17^ ^]^ In this study, we further explored the mechanism by which PBM affects the progression of AD in vivo and in vitro by regulating microglial phenotype switching.

Exosomes are small vesicles measuring 40–150 nm in size. They have been suggested to play a significant role in the pathogenesis and progression of AD. The data indicate that extracellular vesicles could be involved in the brain's inflammatory response in AD .^[^ ^18^ ^]^ Currently, researchers are focusing on microglia‐mediated neuroinflammation and its possible link to the etiology of AD through exosomes.^[^ ^19^, ^20^ ^]^ Ruan et al. demonstrated that P2RX7‐induced exosome secretions from microglia spread tau pathology and enhanced inflammatory cytokine gene expression in AD mice.^[^ ^21^ ^]^ Gao et al. found that glutaminase C overexpression in mouse microglia increased exosome release, including specific packaging of pro‐inflammatory microRNAs (miRNAs) that promoted the establishment of an inflammatory microenvironment.^[^ ^22^ ^]^ However, the relationship between the contents of exosomes and microglial phenotype changes, as well as their effect on AD, remain unclear.

In this study, we hypothesized that 1070‐nm light pulsed at 10 Hz could modulate microglia to switch from the M1 to M2 phenotype and promote the secretion of exosomes containing anti‐inflammatory miRNAs, which could be taken up by neurons to reduce neuroinflammation and promote dendritic spine plasticity, resulting in improved cognitive deficits in 5xFAD mice. Our results showed that 1070‐nm light pulsed at 10 Hz induced M2 microglia phenotypes that secreted exosomes containing miR‐7670‐3p targeting activating transcription factor 6 (ATF6), a key regulator of endoplasmic reticulum (ER) stress, during the unfolded protein response (UPR). ER stress results from accumulation of unfolded proteins in the ER. Previous studies suggested that increased ER stress in neurons was associated with AD development.^[^ ^23^, ^24^, ^25^ ^]^ ER stress has been found to promote inflammation in AD.^[^ ^26^ ^]^ We found that miR‐7670‐3p downregulated ATF6 expression, thereby reducing ER stress and inflammation in 5xFAD mice. As a result, it maintained the integrity of dendritic spines, ultimately improving cognitive performance.

This study emphasizes the crucial role of exosomes derived from 1070‐nm light‐modulated microglia in the treatment of mice with AD. This finding potentially offers a theoretical foundation for employing near‐infrared PBM as a treatment for AD.

## 2 Results

### 2.1 An Apparatus for Irradiating Cells with 1070‐nm Light was Developed

**Figure 1:**
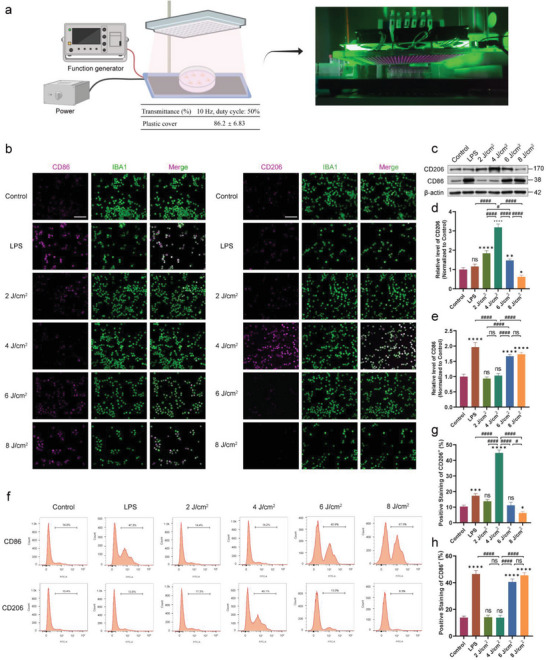
Exposure to 1070‐nm light promoted M2 polarization and inhibited M1 polarization in BV2 cells. a) The irradiating apparatus and transmittance of 1070‐nm light (image created with Biorender.com). b) Immunofluorescence staining shows the expression of M1 marker CD86 and M2 marker CD206 after treating BV2 cells with saline, lipopolysaccharide (LPS). or different fluences of 1070‐nm light at 10 Hz for 24 h. Bar = 100 µm. c–e) Western blotting analysis of BV2 cells treated with saline, LPS, or different fluences of 1070‐nm light at 10 Hz for 24 h shows expression of CD86 and CD206. f–h) Flow cytometry analysis of BV2 cells with different treatments after 24 h shows expression of CD86 and CD206. LPS was used as a positive control for M1 polarization. The results were based on three repeated experiments. Data are presented as mean ± SD. ^*^p < 0.05, ^**^p < 0.01, ^***^p < 0.001, and ^****^p < 0.0001 versus the control group; *^#^p* < 0.05, *^##^p* < 0.01, *^###^p* < 0.001, and *^####^p* < 0.0001 versus the indicated group.

An apparatus was developed to irradiate cells with 1070‐nm light (**Figure** **1**a). Light pulses were set at 10 Hz (duty cycle: 50%) to match the frequency of a previous animal irradiation apparatus.^[^ ^16^ ^]^ A wavelength of 1070 ± 50 nm was chosen for the LED array, with an average power density of 12 mW cm^−2^. Approximately 86.2% of the incident 1070‐nm light penetrated through the lid of the cell culture dish. Consequently, the incident irradiance of 12 mW cm^−2^ at 10 Hz with a 50% duty cycle resulted in an irradiance of ≈5.2 mW cm^−2^ on cells.

### 2.2 Exposure to 1070‐nm Light Promoted M2 Polarization and Inhibited M1 Polarization in Microglia

As shown in Figure 1a, to test the effects and determine the optimal irradiance of 1070‐nm light on BV2 cells, cells received treatments of 2, 4, 6, or 8 J cm^−2^ of 1070‐nm light pulsed at 10 Hz. Figure 1b shows basal staining of CD86 and CD206 in controls, suggesting basal activation. Compared to controls, LPS upregulated M1 phenotype activation by intensely staining CD86 and lightly staining CD206 at 24 h (Figure 1b). After 4 J cm^−2^ treatment for 24 h, CD206 expression markedly increased compared to the control, LPS, 2, 6, and 8 J cm^−2^ groups (Figure 1b–d). Flow cytometry was used to quantify the M1/M2 phenotype post‐treatment (Figure 1f–h), and the results were consistent with immunofluorescence and Western blotting. LPS decreased the number of CD206^+^ cells and increased the number of CD86^+^ cells. Conversely, 1070‐nm at 4 J cm^−2^ increased the number of CD206^+^ cells and decreased the number of CD86^+^ cells; however, 2 J cm^−2^ had no effect (Figure 1f–h). Notably, 6 J cm^−2^ and 8 J cm^−2^ of 1070‐nm light significantly elevated CD86 expression and the number of CD86^+^ cells (Figure 1b,c,e,f,h). Concurrently, cellular viability was noticeably diminished in the 6 J cm^−2^ and 8 J cm^−2^ versus 4 J cm^−2^ and 2 J cm^−2^ groups (Figure S1, Supporting Information). Hence, 2 and 4 J cm^−2^ were selected for further study.

### 2.3 Exosome Isolation and Identification

**Figure 2:**
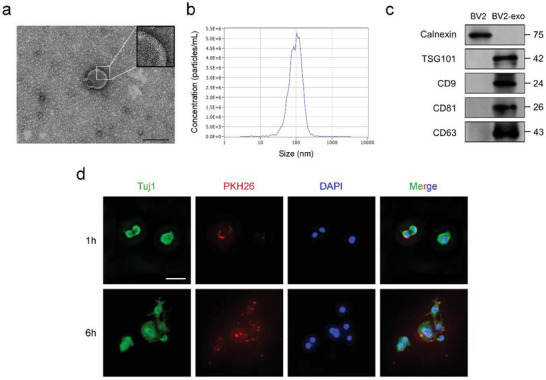
Characterization of exosomes released from BV2 cells subjected to 4 J cm^−2^ of 1070‐nm light at 10 Hz. a) Representative transmission electron microscope (TEM) images of BV2‐exosomes (BV2‐exos). Magnification shows the membrane‐limited particles. Bar = 100 nm. b) Nanoparticle tracking analysis (NTA) of BV2‐exos. c) Western blotting analysis of protein markers of exosomes. d) Uptake of PKH26‐stained exosomes in N2a/APP695swe cells detected by fluorescence microscopy. Scale bar = 50 µm.

To determine whether M2 microglia induced by 1070‐nm light inhibited neuroinflammation in AD via secreted exosomes, we isolated exosomes from the supernatant of BV2 cells treated with 4 J cm^−2^ 1070‐nm light. The transmission electron microscope (TEM) images revealed membrane‐limited particles that were homogeneous in appearance and the diameter ranged from 50 to 120 nm (**Figure** **2**a). The nanoparticle tracking analysis (NTA) showed particles of 110 nm with a homogeneous population and low dispersion, as indicated by a low polydispersity index of 0.19 ± 0.08. (Figure 2b). Western blot analysis revealed the isolated particles contained exosome markers (TSG101, CD9, CD81, and CD63) (Figure 2c). To verify exosome internalization by N2a/APP695swe cells, exosomes from 1070‐nm light‐treated BV2 cells were labeled with PKH26. Administration of PKH26‐labeled exosomes to N2a/APP695swe cells led to significant uptake of red fluorescence after 1 h, and further increased exosome uptake after 6 h (Figure 2d).

### 2.4 Eoxsomes Derived from 1070‐nm Light‐Treated BV2 Cells Protected N2a/app695swe Cells Against Inflammation and Aβ Accumulation

**Figure 3:**
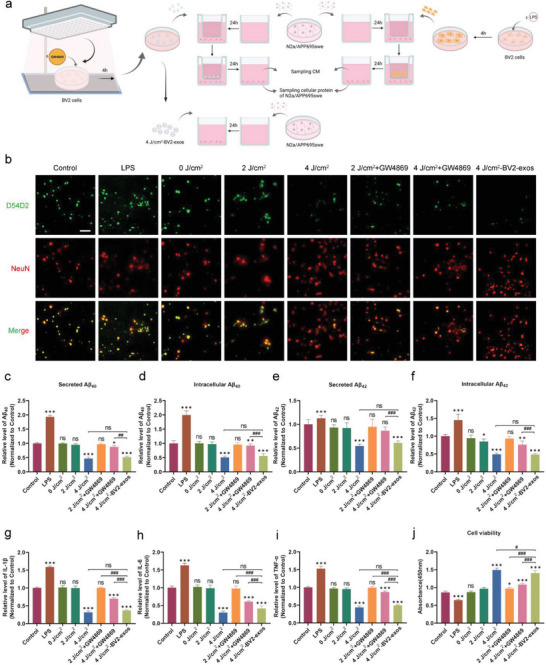
Effects of exosomes derived from 1070‐nm light‐treated BV2 cells on inflammation and attenuation of Aβ deposition. a) Experimental schematic of 1070‐nm light irradiation and exosome treatment (image created with Biorender.com). b) immunofluorescence staining for Aβ with D54D2 in N2a/APP695swe cells. Bar = 100 µm. c,d) Secreted Aβ_40_ and intracellular Aβ_40_ of N2a/APP695swe cells were determined by ELISA. e,f) Secreted Aβ_42_ and intracellular Aβ_42_ were determined by ELISA. g–i) Levels of the inflammatory cytokines IL‐1β, IL‐6, and TNF‐α in N2a/APP695swe cell supernatant from the different groups were measured. j) Cell viability was detected by a CCK8 viability assay 24 h after treatment. For the M1 polarization positive control, LPS was incubated with BV2 cells. The results were based on three repeated experiments. Data are presented as mean ± SD. ^*^p < 0.05, ^**^p < 0.01, ^***^p < 0.001, and ^****^p < 0.0001 versus the control group; *^#^p* < 0.05, *^##^p* < 0.01, *^###^p* < 0.001, and *^####^p* < 0.0001 versus the indicated group.

To determine the effects of exosomes from 1070‐nm light‐treated BV2 cells on N2a/APP695swe cells, we used a Transwell co‐culture system with 0.4‐µm pores between cells. A contact co‐culture system created exosomes with N2a/APP695swe cells (**Figure** **3**a). BV2 cells were pretreated with GW4869, which inhibits exosome secretion. As expected, immunofluorescence and ELISA showed that LPS induced high Aβ concentrations in N2a/APP695swe cells (Figure 3b–f). In contrast, the Aβ burden decreased significantly in the 4 and 4 J cm^−2^‐BV2‐exosomes (4 J cm^−2^‐BV2‐exos) groups. However, no significant difference was found between the control, 0, 2, or 2 J cm^−2^ + GW4869 groups in Aβ burden. We assessed IL‐6, IL‐1β, and TNF‐α concentrations (Figure 3g–i). The concentrations were significantly reduced in the 4 J cm^2^‐BV2‐exos and 4 J cm^−2^ groups. Consistently, the 4 J cm^−2^‐BV2‐exos and 4 J cm^−2^ 1070‐nm light treatment groups showed comparatively increased cell viability (Figure 3j). The protective effects, however, were inhibited after GW4869 pretreatment. Notably, we also observed that the Aβ concentration and level of inflammation were partially reduced in the 4 J cm^−2^ + GW4869 group when compared with the control group. This can be explained by other cytokines that may be secreted by 1070‐nm light‐treated BV2 cells that have an anti‐inflammatory effect. These findings indicated that exosomes derived from 4 J cm^−2^ 1070‐nm light‐treated BV2 cells protected the N2a/APP695swe cells against inflammation and Aβ accumulation.

### 2.5 Exosomes Derived from 1070‐nm Light‐Treated BV2 Cells Prompted Neurite Growth in N2a/APP695swe cells

**Figure 4:**
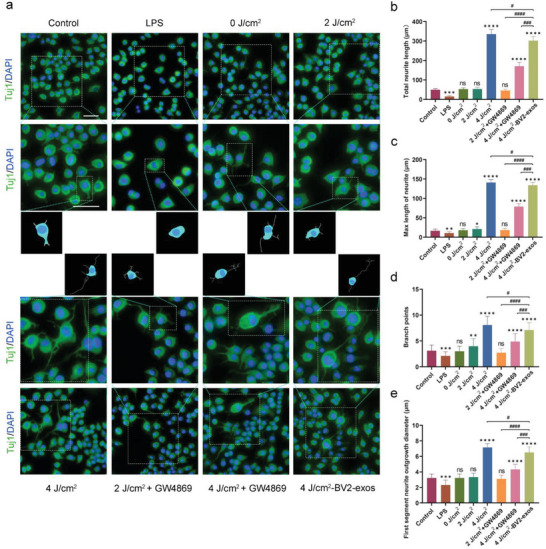
Effects of exosomes derived from 1070‐nm light‐treated BV2 cells on neurite outgrowth in N2a/APP695swe cells. a) Representative immunofluorescence images and morphology schematics of neurite outgrowth in N2a/APP695swe cells of different treatment groups. Scale bar = 50 µm. The box shows magnified and outlined N2a/APP695swe cells in each group. Scale bar = 50 µm. b–e) Four structural plasticity parameters were measured: total length, maximum length of neurites, branch points, and the first segment neurite outgrowth diameter. For the M1 polarization positive control, LPS was incubated with BV2 cells. The results were based on three repeated experiments. Data are presented as mean ± SD. ^*^p < 0.05, ^**^p < 0.01, ^***^p < 0.001, and ^****^p < 0.0001 versus the control group; *^#^p* < 0.05, *^##^p* < 0.01, *^###^p* < 0.001, and *^####^p* < 0.0001 versus the indicated group.

To determine whether 4 J cm^−2^‐BV2‐exos treatment promoted neurite outgrowth, we measured the total length, maximum length, branch points, and diameter of the first neurite segment extending from Tuj1‐stained N2a/APP695swe cells. We found that N2a/APP695swe cells from the 4 J cm^−2^‐BV2‐exos and 4 J cm^−2^ light treatment groups exhibited radially extended and branched neurites. However, the total length, maximum length, branch points, and diameter were significantly reduced in GW4869‐pretreated cells compared to the 4 J cm^−2^‐BV2‐exos and 4 J cm^−2^ light treatment groups (**Figure** **4**a–e). Similarly, 2 J cm^−2^ of 1070‐nm light did not enhance neurite growth. Overall, the data indicated exosomes from 4 J cm^−2^ 1070‐nm light‐treated BV2 cells provided neuroprotective effects by promoting neurite growth.

### 2.6 RNA‐Seq Analysis of Exosomal Small RNAs from the 1070‐nm Treated Group and Control Group and Bioinformatics Analysis of Potential Target Genes

**Figure 5:**
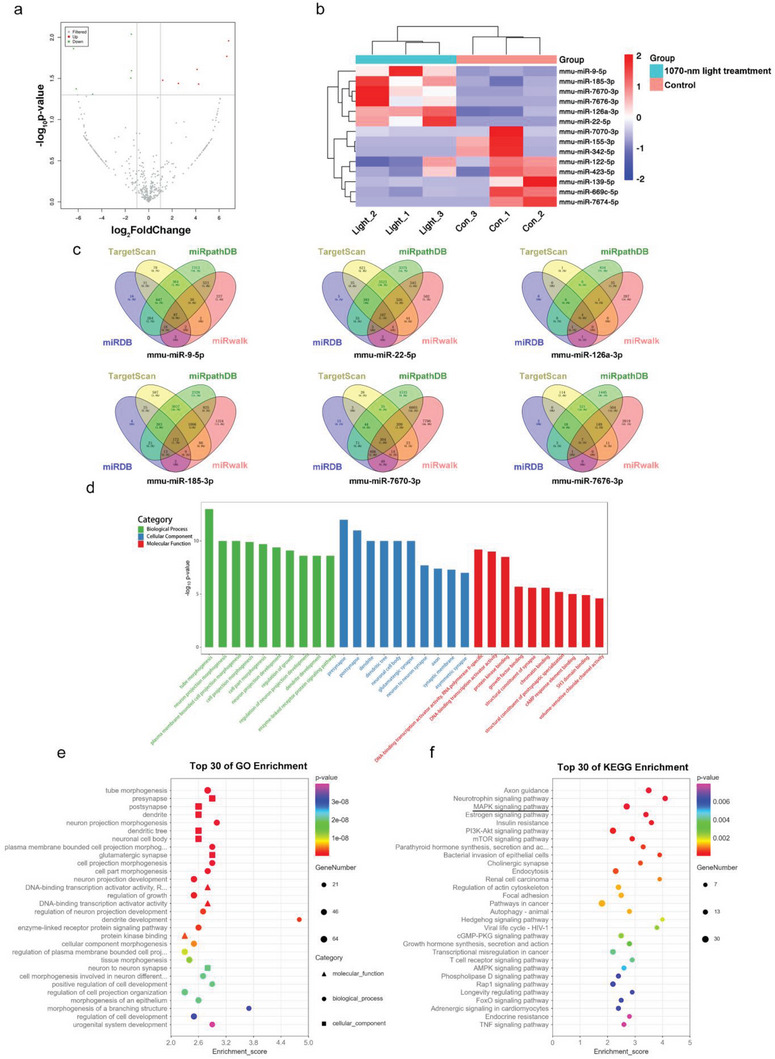
RNA‐seq analysis of exosomal miRNAs and analysis of potential target genes. a,b) Volcano plot and heatmap for differentially expressed miRNA identified in BV2‐exos from 4 J cm^−2^ 1070‐nm light‐treated and control groups. The red and green dots represent significant miRNAs with |log_2_fold change| >1 and *p*‐value <0.05, and the grey dots represent non‐significant results. c) The overlapped target genes of the top six miRNAs were predicted with four online tools (TargetScan, miRDB, miRWalk, and miRpathDB). d) Gene ontology (GO) enrichment of the predicted target genes. e) The top 30 terms from the GO enrichment analysis. f) The top 30 terms from the Kyoto Encyclopedia of Genes and Genomes (KEGG) enrichment analysis.

We further examined the small RNAs expression in exosomes derived from 4 J cm^−2^ 1070‐nm light‐treated BV2 cells and the control group by high throughput RNA sequencing. The results showed that compared to miRNAs, the expression levels of tRNA, rRNA and snRNA were relatively low (Figure S2, Supporting Information). Therefore, exosomal miRNAs were suspected to play a crucial role in the process of 1070‐nm light treatment. A large number of exosomal miRNAs were identified, and the fold changes for the miRNAs with significant differential expression are shown in **Figure** **5**a,b. The top six overexpressed miRNAs were mmu‐miR‐9‐5p, mmu‐miR‐185‐3p, mmu‐miR‐7676‐3p, mmu‐miR‐7670‐3p, mmu‐miR‐126a‐3p, and mmu‐miR‐22‐5p. The intersection of miRNA target genes was screened using four different online databases (TargetScan, miRDB, miRWalk, and miRpathDB), and a total of 681 potential target genes were identified (Figure 5c). In Gene Ontology (GO) enrichment analysis, the five neuronal growth‐related terms “tube morphogenesis”, “presynapse”, “postsynapse”, “dendrite”, and “neuron projection morphogenesis” were detected (Figure 5d,e). In the Kyoto Encyclopedia of Genes and Genomes (KEGG) enrichment analysis, seven pathways related to inflammation and programmed cell death were identified, including “MAPK signaling pathway”, “PI3K‐Akt signaling pathway”, “mTOR signaling pathway”, “autophagy”, “T cell receptor signaling pathway”, “FoxO signaling pathway”, and “TNF signaling pathway” (Figure 5f).

### 2.7 mmu‐miR‐7670‐3p Inhibits the Expression of ATF6 in N2a/APP695swe Cells

**Figure 6:**
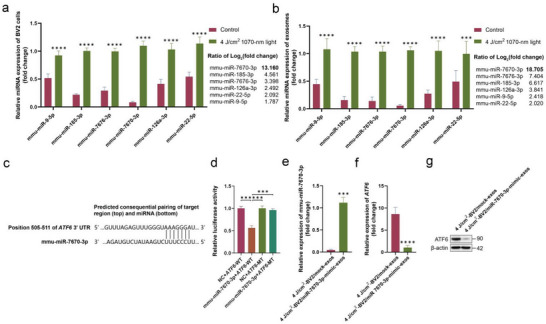
mmu‐miR‐7670‐3p is highly expressed in exosomes derived from 1070‐nm light‐treated BV2 cells and targets ATF6. a,b) Quantitative real‐time PCR (qRT‐PCR) analysis detected the expression level of mmu‐miR‐9‐5p, mmu‐miR‐185‐3p, mmu‐miR‐7670‐3p, mmu‐miR‐7676‐3p, mmu‐miR‐126a‐3p, and mmu‐miR‐22‐5p in BV2 cells and exosomes derived from the 4 J cm^−2^‐1070‐nm light‐treated group and non‐light‐treated group. Data showing miRNA expression fold changes compared to those of the control group are presented. c) The predicted binding sites of mmu‐miR‐7670‐3p to *ATF6*. d) Dual luciferase reporter assays of 293T cells cotransfected with mmu‐miR‐7670‐3p‐mimics and the luciferase reporter plasmid that was inserted with the wild‐type (WT) or mutated (MT)‐*ATF6* 3′ UTR. Data are mean ± SD, n = 3. e) qRT‐PCR results showing the relative expression of mmu‐miR‐7670‐3p in N2a/APP695swe cells after being treated with 4 J cm^−2^‐BV2/miR‐7670‐3p‐mimic‐exos or 4 J cm^−2^‐BV2/mock‐exos. f) qRT‐PCR showing the relative mRNA expression of *ATF6* in N2a/APP695swe cells treated with 4 J cm^−2^‐BV2/miR‐7670‐3p‐mimic‐exos or 4 J cm^−2^‐BV2/mock‐exos. g) The protein levels of ATF6 in N2a/APP695swe cells were tested by western blotting. The results were based on three repeated experiments. ^*^p < 0.05, ^**^p < 0.01, ^***^p < 0.001, and ^****^p < 0.0001 versus the control group; *^#^p* < 0.05, *^##^p* < 0.01, *^###^p* < 0.001, and *^####^p* < 0.0001 versus the indicated group.

We investigated the differential expression of miRNAs in BV2 cells and exosomes between the 4 J cm^−2^ 1070‐nm light‐treated and control groups using qRT‐PCR. We found that the most differentially expressed miRNAs in the 4 J cm^−2^ exosomes showed negligible differences in cells (**Figure** **6**a,b). Taking these results into consideration, we suspect that the miRNAs in microglial cells or exosomes may be actively selected for secretion into the microenvironment under the regulation of 4 J cm^−2^ light. To verify the hypothesis, we chose mmu‐miR‐7670‐3p, the most significantly differentially expressed miRNA in both BV2 cells and BV2‐exos, for further investigation.

Mmu‐miR‐7670‐3p is predicted to bind to *ATF6* (Figure 6c), which is known to participate in the regulation of ER stress and neuroinflammation in AD, affecting Aβ accumulation and neuronal synaptic function. The evidence shows that ATF6 might have cross‐talk with the mitogen‐activated protein kinase (MAPK) signaling pathway in regulating inflammation.^[^ ^27^, ^28^, ^29^ ^]^ To investigate the *ATF6* association with mmu‐miR‐7670‐3p, we first detected mmu‐miR‐7670‐3p‐targeted *ATF6* regulation using a luciferase reporter. We found that mmu‐miR‐7670‐3p downregulated expression of the luciferase reporter containing the *ATF6* 3′ UTR (Figure 6d). To further explore whether mmu‐miR‐7670‐3p regulated the expression of ATF6, we transfected mmu‐miR‐7670‐3p‐mimics or mock‐mimics into 4 J cm^−2^ light‐treated BV2 cells and collected their exosomes for treating N2a/APP695swe cells to detect the expression of ATF6. The overexpression of mmu‐miR‐7670‐3p markedly decreased the expression of ATF6 in N2a/APP695swe cells (Figure 6e–g).

### 2.8 mmu‐miR‐7670‐3p Attenuated the Production of the Aβ Peptide and Suppressed the Inflammatory Response to Regulate Neurite Outgrowth in N2a/APP695swe Cells

**Figure 7:**
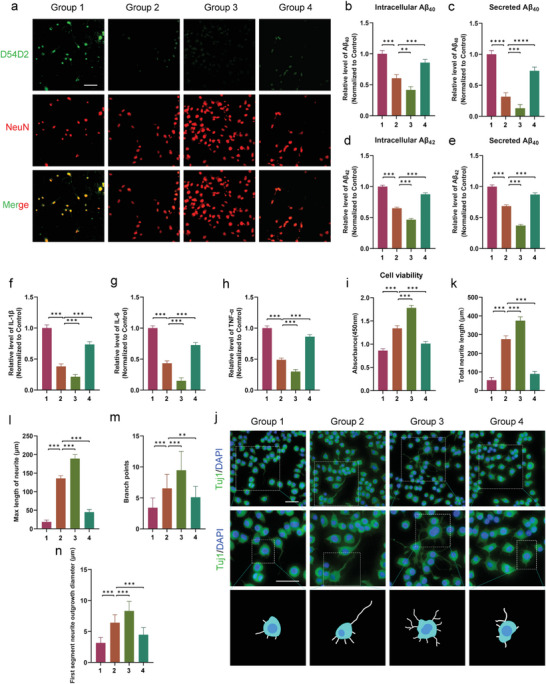
mmu‐miR‐7670‐3p actively attenuated inflammation and Aβ peptide levels, prompting neurite outgrowth. a) D54D2 immunofluorescence staining of Aβ in N2a/APP695swe cells 24 h after treatment with mock exosomes from 4 J cm^−2^ 1070‐nm light‐treated BV2 cells, or exosomes from 4 J cm^−2^ light‐treated BV2 cells loaded with mmu‐miR‐7670‐3p mimics or an inhibitor. Bar = 50 µm. b–e) Secreted and intracellular Aβ40 and Aβ42 in N2a/APP695swe cells determined by ELISA. f–h) Levels of inflammatory cytokines IL‐1β, IL‐6, and TNF‐α in N2a/APP695swe cell supernatants measured at 24 h. i) Cell viability detected using a CCK8 assay at 24 h post‐treatment. j) Representative immunofluorescence images and neurite outgrowth morphology schematics of N2a/APP695swe cells in different groups. Scale bar = 50 µm. The box shows magnified and outlined N2a/APP695swe cells in each group. Scale bar = 50 µm. k–n) Four structural plasticity parameters were measured: total length, maximum length, branch points, and first segment neurite outgrowth diameter. The results were based on three repeated experiments. Group 1, control group; Group 2, 4 J cm^−2^‐BV2/mock‐exos group; Group 3, 4 J cm^−2^‐BV2/mmu‐miR‐7670‐3p‐mimic‐exos group; Group 4, 4 J cm^−2^‐BV2/mmu‐miR‐7670‐3p‐inhibitor‐exos group. Data are presented as mean ± SD. ^*^p < 0.05, ^**^p < 0.01, ^***^p < 0.001, and ^****^p < 0.0001 versus the control group; *^#^p* < 0.05, *^##^p* < 0.01, *^###^p* < 0.001, and *^####^p* < 0.0001 versus the indicated group.

To explore the impact of exosomal mmu‐miR‐7670‐3p on N2a/APP695swe cells, mmu‐miR‐7670‐3p mimics were transfected into 4 J cm^−2^ 1070‐nm light‐treated BV2 cell exosomes, while an inhibitor was used to block mmu‐miR‐7670‐3p in 4 J cm^−2^ 1070‐nm light‐treated BV2 cell exosomes. We detected whether exosomal mmu‐miR‐7670‐3p attenuated the production of the Aβ peptide. As shown in **Figure** **7**a–e, treatment with 4 J cm^−2^‐BV2/mmu‐miR‐7670‐mimic‐exos dramatically attenuated Aβ peptide intracellular levels and secretions (Figure 7a–e). Compared to the 4 J cm^−2^‐BV2/mock‐exos treatment, mmu‐miR‐7670‐3p mimic exosomes decreased inflammatory factor concentrations in N2a/APP695swe cell supernatants (Figure 7f–h). Conversely, mmu‐miR‐7670‐3p inhibitor exosome treatment dampened anti‐inflammatory effects and decreased N2a/APP695swe cell viability (Figure 7i). Additionally, we determined the effects of mmu‐miR‐7670‐3p on N2a/APP695swe cell neurite outgrowth. The total length, maximum length, branch points, and first segment diameter significantly increased with mmu‐miR‐7670‐3p mimic exosomes (Figure 7j–n). However, these promoting effects were suppressed by mmu‐miR‐7670‐3p inhibitor exosomes. In summary, these findings indicated that exosomal mmu‐miR‐7670‐3p protected N2a/APP695swe cells against inflammation, ameliorated Aβ generation, and enhanced neurite growth.

### 2.9 Exosomes Derived from 1070‐nm Light‐Treated Microglia Improved Spatial Learning and Memory Abilities in 5xFAD mice

**Figure 8:**
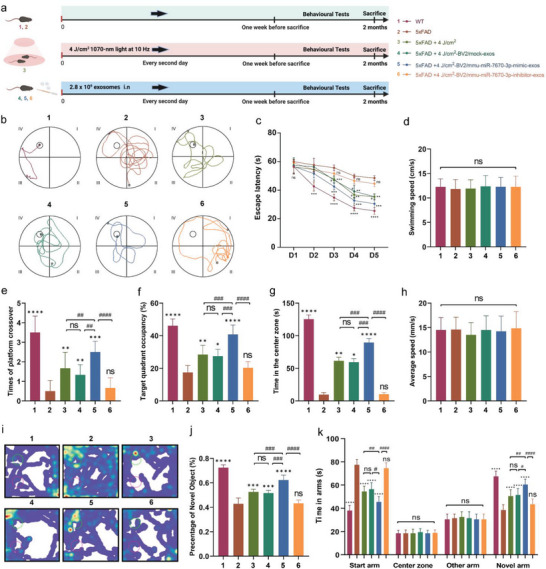
Exosomal mmu‐miR‐7670‐3p derived from 1070‐nm light‐treated microglia improved spatial learning and memory ability in 5xFAD mice. a) Schematic illustrating the chronological sequence of treatments, Morris water maze (MWM), Y‐maze, open field, and object recognition testing (image created with Biorender.com). b) Results of the typical escape route of WT and control mice, and those treated with 4 J cm^−2^ 1070‐nm light, exosomes derived from 4 J cm^−2^ 1070‐nm light‐treated BV2 cells, and exosomes from 4 J cm^−2^ 1070‐nm light treating BV2 cells loaded with mmu‐miR‐7670‐3p mimics or inhibitor groups, that were cognitively tested using the MWM, including the escape latencies (c), swimming speed (d), platform crossover (e), and swimming time in the target quadrant (f).,g,h) Open field test, including the average speed and time in the center zone. i,j) The object recognition test, including the typical heatmap and percentage of mice that stayed with a novel object. k) The Y‐maze test. Group 1, WT; Group 2, 5xFAD; Group 3, 5xFAD + 4 J cm^−2^; Group 4, 5xFAD + 4 J cm^−2^‐BV2/mock‐exos; Group 5, 5xFAD + 4 J cm^−2^‐BV2/mmu‐miR‐7670‐3p‐mimic‐exos; Group 6, 5xFAD + 4 J cm^−2^‐BV2/mmu‐miR‐7670‐3p‐inhibitor‐exos. Data are presented as mean ± SD, n = 6 per group. ^*^p < 0.05, ^**^p < 0.01, ^***^p < 0.001, and ^****^p < 0.0001 versus the control group; *^#^p* < 0.05, *^##^p* < 0.01, *^###^p* < 0.001, and *^####^p* < 0.0001 versus the indicated group.

To test the effects and the potential mechanism of exosomes derived from 4 J cm^−2^ 1070‐nm light‐treated BV2 cells on 5xFAD mice, mice were treated with 4 J cm^−2^ 1070‐nm light, exosomes derived from 4 J cm^−2^ 1070‐nm light‐treated BV2 cells, and exosomes from 4 J cm^−2^ 1070‐nm light‐treated BV2 cells loaded with mmu‐miR‐7670‐3p mimics or inhibitor every two days before behavioral tests, for a total of 26 times. Two months after the treatment, mice were tested in a series of behavioral tests for spatial learning and memory ability, including the Morris water maze (MWM), open field, object recognition, and Y‐maze tests (**Figure** **8**a). The mock‐BV2‐exos, 1070‐nm light and mmu‐miR‐7670‐3p‐mimic‐BV2‐exos groups showed immediate and oriented escape paths (Figure 8b). In the MWM, the 4 J cm^−2^‐BV2/mmu‐miR‐7670‐3p‐mimic‐exos group exhibited a shorter escape latency, more crossovers, and longer target quadrant occupancy versus the control, 4 J cm^−2^ light, 4 J cm^−2^‐BV2/mock‐exos, and 4 J cm^−2^‐BV2/mmu‐miR‐7670‐3p‐inhibitor‐exos groups on days 3–5, but with no differences in swim velocity (Figure 8c–f). In open field tests, the 4 J cm^−2^‐BV2/mmu‐miR‐7670‐3p‐mimic‐exos, 4 J cm^−2^ light, and 4 J cm^−2^‐BV2/mock‐exos groups stayed longer in the central area, but with no difference in speeds compared to controls (Figure 8g,h). In object recognition and Y‐maze tests (Figure 8i–k), the 4 J cm^−2^‐BV2/mmu‐miR‐7670‐3p‐mimic‐exos, 4 J cm^−2^ light, and 4 J cm^−2^‐BV2/mock‐exos groups spent more time in novel arms and exploring new objects compared to the other groups. Notably, the 4 J cm^−2^‐BV2/mmu‐miR‐7670‐3p‐mimic‐exos group spent the longest time exploring new objects, suggesting stronger memory retention in 5xFAD mice. These data showed that exosomal mmu‐miR‐7670‐3p improved cognitive function in AD mice.

### 2.10 Promotion of Polarization of Microglia from M1 to M2 In Vivo by 1070‐nm Light

**Figure 9:**
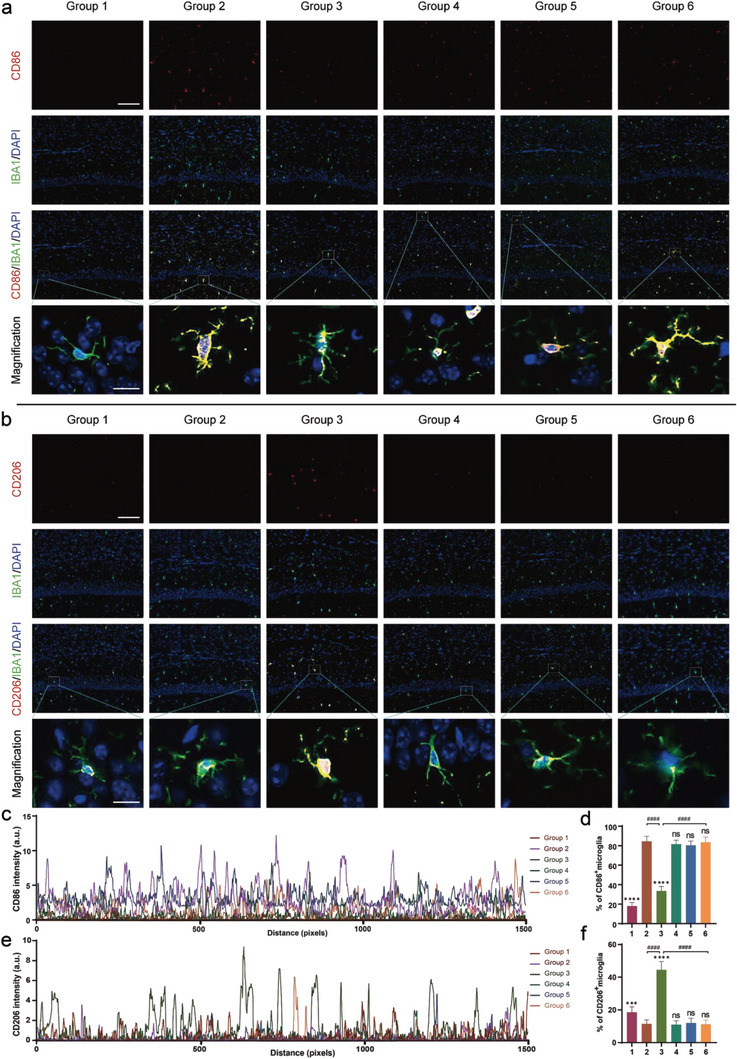
Exposure to 1070‐nm light promotes polarization of microglia from M1 to M2 in vivo. a) Representative immunofluorescent staining of CD86 with IBA1 in the cortex and CA1 regions of the hippocampus (HPC). b) Representative immunofluorescent staining of CD206 with IBA1 in the cortex and CA1 regions. c) Cross‐sectional fluorescence intensity profile of CD86 in mice receiving different treatments. d) Percentages of CD86+ microglial cells in the cortex and CA1 regions. e) Cross‐sectional fluorescence intensity profile of CD206 in mice receiving different treatments. f) Percentages of CD206+ microglial cells in the cortex and CA1 regions. Scale bar = 100 µm for images in their original size; scale bar = 25 µm for magnified images. Group 1, WT; Group 2, 5xFAD; Group 3, 5xFAD + 4 J cm^−2^; Group 4, 5xFAD + 4 J cm^−2^‐BV2/mock‐exos; Group 5, 5xFAD + 4 J cm^−2^‐BV2/mmu‐miR‐7670‐3p‐mimic‐exos; Group 6, 5xFAD + 4 J cm^−2^‐BV2/mmu‐miR‐7670‐3p‐inhibitor‐exos. N = 12 fields of view from six mice per group for (d) and (f). Data are presented as mean ± SD, n = 6 per group. *p < 0.05, **p < 0.01, ***p < 0.001, and ****p < 0.0001 versus the 5xFAD group; #p < 0.05, ##p < 0.01, ###p < 0.001, and ####p < 0.0001 versus the indicated group.

We evaluated the polarization of microglia in the cortex and CA1 regions of both 5xFAD and WT mice using representative markers for M1 (CD86) and M2 (CD206) via double immunofluorescent staining with IBA1, as shown in **Figure** **9**. Notably, there was no substantial difference in the fluorescence density of CD86 or the percentage of CD86^+^ microglial cells between the control group, 4 J cm^−2^‐BV2/mock‐exos group, mmu‐miR‐7670‐3p‐mimic‐BV2‐exos group, or mmu‐miR‐7670‐3p‐inhibitor‐BV2‐exos group. However, in comparison with the aforementioned groups, the CD86 fluorescence density and proportion of CD86^+^ microglial cells substantially declined in 5xFAD mice who underwent 4 J cm^−2^ light exposure, as demonstrated in Figure 9c,d. Meanwhile, we observed no significant difference in the CD206 fluorescence density or the proportion of CD206^+^ microglial cells between the control group, 4 J cm^−2^‐BV2/mock‐exos group, mmu‐miR‐7670‐3p‐mimic‐BV2‐exos group, and mmu‐miR‐7670‐3p‐inhibitor‐BV2‐exos group (Figure 9e,f). Nevertheless, 5xFAD mice treated with 4 J cm^−2^ light experienced a higher CD206 fluorescence density and a greater proportion of CD206^+^ microglial cells. These experimental outcomes suggest that within the brain of 5xFAD mice, 1070‐nm light at 4 J cm^−2^, rather than exosomes, promoted the polarization of microglial cells from M1 to M2. The exosomes derived from 4 J cm^−2^ light‐treated microglia did not appear to regulate the phenotypic changes of microglia in vivo.

### 2.11 Exosomes Derived from 1070‐nm Light‐Treated Microglia Attenuated Aβ Peptide Levels and Neuroinflammation in 5xFAD Mice

To investigate if BV2‐exos in vivo targeted pathological areas in 5xFAD mice, we labeled BV2‐exos with PKH26 prior to intranasal administration. After 24 h, brain sections underwent immunofluorescence staining. We observed colocalization of PKH26 particles and NeuN in the cortex and HPC of 5xFAD mice, indicating that BV2‐exos efficiently integrated into neurons in these regions following intranasal administration (Figure S3, Supporting Information).

**Figure 10:**
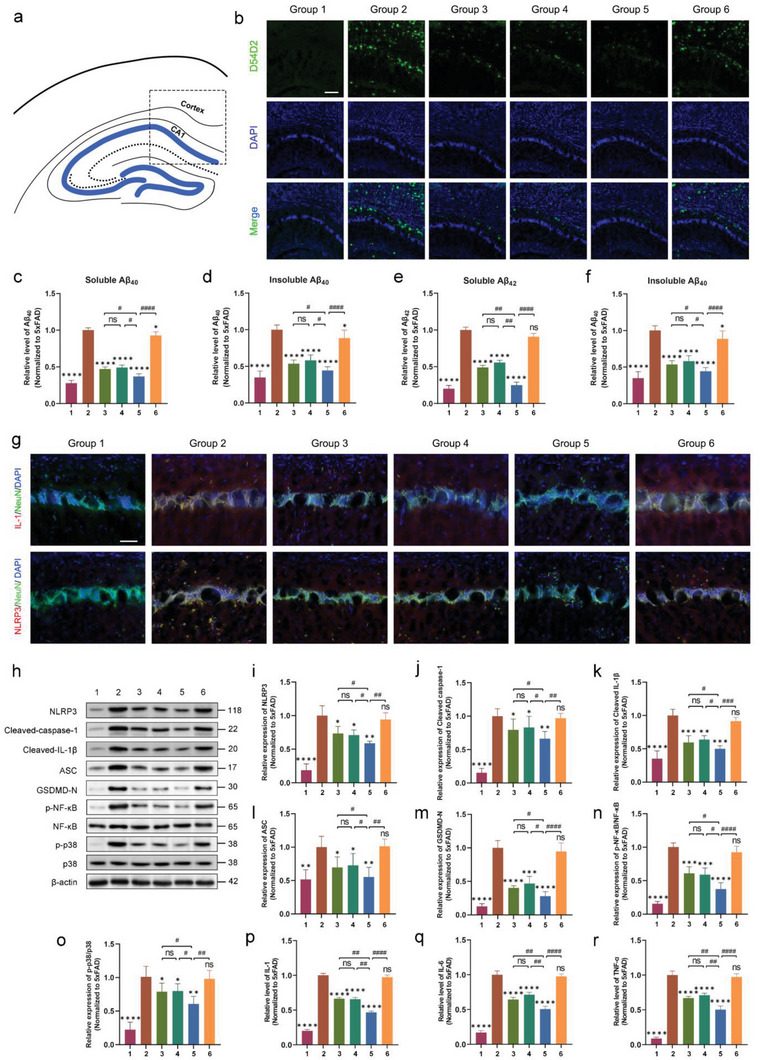
Exosomal mmu‐miR‐7670‐3p derived from 1070‐nm light‐treated microglia alleviated Aβ deposition and inflammation in 5xFAd mice. a) Schematic shows the Aβ stained area of the cortex and CA1. b) Brain slices of the cortex or HPC from mice at 6 months (6 M) were stained with anti‐Aβ (D54D2, green). Scale bar = 200 µm. c–f) Soluble and insoluble forms of Aβ40 and Aβ42 in the brain tissue of mice at 6 M were determined by ELISA. g) Co‐localization of NLRP3 or IL‐1β in neurons of the cortex and CA1 suggesting that levels of the NLRP3 inflammasome and IL‐1β decreased after treatment with exosomes derived from 4 J cm^−2^ light‐treated BV2 cells loaded with mmu‐miR‐7670‐3p mimics. Scale bar = 100 µm. h–o) Western blotting and quantitative analysis of NLRP3, cleaved caspase‐1, cleaved IL‐1β, ASC, GSDMD‐N, p‐NF‐κB, NF‐κB, p‐p38, and p38 protein in brain extracts of the cortex and CA1. p–r) Levels of the inflammatory cytokines IL‐1β, IL‐6, and TNF‐α in brain tissue were measured by ELISA. Group 1, WT; Group 2, 5xFAD; Group 3, 5xFAD + 4 J cm^−2^; Group 4, 5xFAD + 4 J cm^−2^‐BV2/mock‐exos; Group 5, 5xFAD + 4 J cm^−2^‐BV2/mmu‐miR‐7670‐3p‐mimic‐exos; Group 6, 5xFAD + 4 J cm^−2^‐BV2/mmu‐miR‐7670‐3p‐inhibitor‐exos. Data are presented as mean ± SD, n = 6 per group. ^*^p < 0.05, ^**^p < 0.01, ^***^p < 0.001, and ^****^p < 0.0001 versus the 5xFAD group; *^#^p* < 0.05, *^##^p* < 0.01, *^###^p* < 0.001, and *^####^p* < 0.0001 versus the indicated group.

We used qRT‐PCR to determine mmu‐miR‐7670‐3p expression in brain tissue. Mmu‐miR‐7670‐3p expression significantly increased in 5xFAD mice treated with 4 J cm^−2^‐BV2/mmu‐miR‐7670‐3p‐mimic‐exos and 4 J cm^−2^ light and 4 J cm^−2^‐BV2/mock‐exos versus controls or 4 J cm^−2^‐BV2/mmu‐miR‐7670‐3p‐inhibitor‐exos (Figure S4, Supporting Information). As expected, mice treated with 4 J cm^−2^‐BV2/mmu‐miR‐7670‐3p‐mimic‐exos showed the highest mmu‐miR‐7670‐3p expression. There was no significant difference between the 4 J cm^−2^ light and 4 J cm^−2^‐BV2/mock‐exos groups. To evaluate exosomes from 4 J cm^−2^‐treated BV2 cells, we examined the Aβ burden in 6 M mice by staining with anti‐Aβ antibody (D54D2) (**Figure** **10**a,b) or by ELISA (Figure 10c–f). The results showed a significant decrease in the Aβ burden in the 4 J cm^−2^‐BV2/mmu‐miR‐7670‐3p‐mimic‐exos, 4 J cm^−2^ 1070‐nm light, and 4 J cm^−2^‐BV2/mock‐exos groups with a decrease in the inflammatory level at 6 M. Moreover, treatment with 4 J cm^−2^‐BV2/mmu‐miR‐7670‐3p‐mimic‐exos showed a lower Aβ burden and lower inflammation compared to the 4 J cm^−2^ light and 4 J cm^−2^‐BV2/mock‐exos groups. However, inhibiting by 4 J cm^−2^‐BV2/mmu‐miR‐7670‐3p‐inhibitor‐exos blocked the effects of attenuating Aβ levels and inflammation. There was no significant difference between the 4 J cm^−2^ light and 4 J cm^−2^‐BV2/mock‐exos groups. The NLR family pyrin domain containing 3 (NLRP3) inflammasome consists of NLRP3, apoptotic speck like protein (ASC), cleaved caspase‐1, and mature IL‐1β, which were significantly elevated in the AD brain, indicating activation of the NLRP3 inflammasome. Additionally, to verify the specific expression of NLRP3 and IL‐1β in neurons, we co‐localized these inflammatory mediators with NeuN. NLRP3 and IL‐1β predominantly co‐localized with NeuN in the cortex and CA1 region of mice (Figure 10g), confirming activation of the inflammatory pathway. In light of the KEGG enrichment analysis results (Figure 5f), the MAPK signaling pathway appears to play a role in regulating the inflammatory response. Notably, nuclear factor kappa‐B (NF‐κB) serves as an essential inflammatory signal downstream of the MAPK signaling pathway. To investigate this, we measured the expression ratios of p‐NF‐κB/NF‐κB and p‐p38/p38 proteins. The results showed a significant increase in the expression ratios of p‐NF‐κB/NF‐κB and p‐p38/p38 in the brain tissues of 5xFAD mice, whereas in the 4 J cm^−2^‐BV2/mmu‐miR‐7670‐3p‐mimic‐exos group, we observed a notable decrease in the expression of these proteins with decreased inflammatory cytokines (Figure 10h,n,o). These findings suggest that mmu‐miR‐7670‐3p has an inhibitory effect on classical inflammatory signaling molecules in 5xFAD.

### 2.12 Exosomes Derived from 1070‐nm Light‐Treated Microglia Protected Dendrites and Synapses of Neurons in 5xFAD Mice

**Figure 11:**
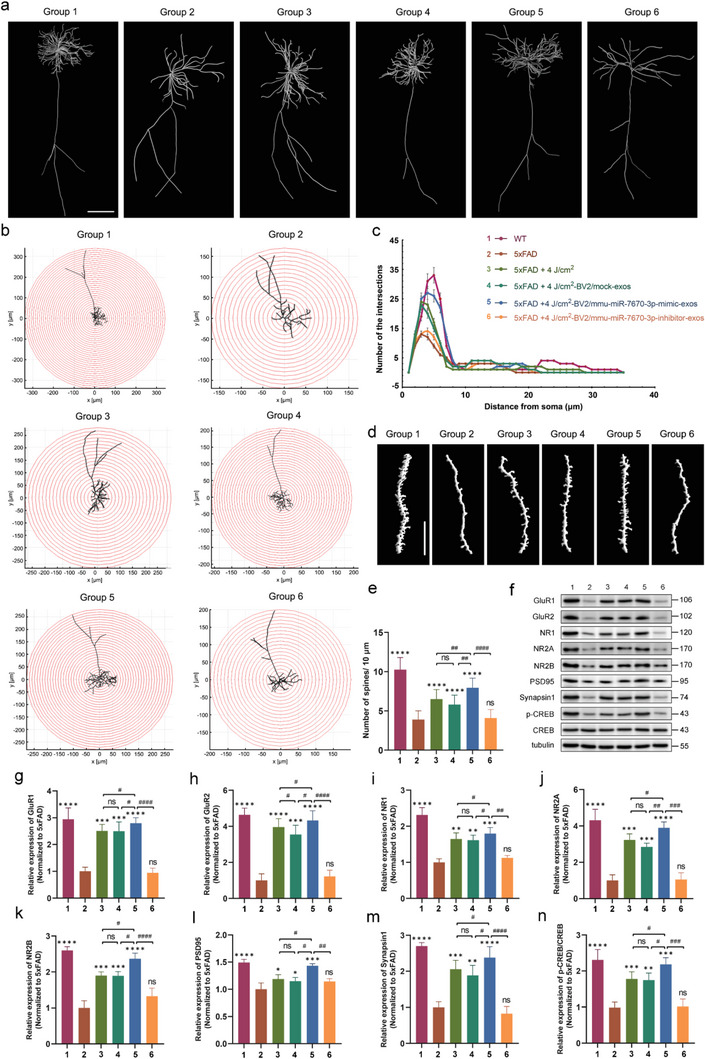
Exosomal mmu‐miR‐7670‐3p protected the synapses and dendrites of neurons in 5xFAD mice. a) Representative 3D reconstructed pyramidal neurons of CA1 with Golgi staining in the different groups. Scale bar = 50 µm. b) Corresponding Sholl analysis of pyramidal neurons from different groups. c) A comparison of the results for the pyramidal neuron for the radial distance at which the maximum number of dendrites are intersected. d) Typical structure of dendrite spines from mice with different treatments e) Quantification of the dendritic spine density in each of the different groups. Six randomly selected neurons from six mice per group were used for dendritic spine analysis. Scale bar = 10 µm. The expression of glutamate receptors and synapsin‐related proteins was determined in specific brain regions of the mice. The brain extracts of the cortex and HPC were used for western blotting (f) and quantitative analysis (g–n) of GluR1, GluR2, NR1, NR2A, NR2B, PSD95, synapsin 1, p‐CREB, and CREB. Group 1, WT; Group 2, 5xFAD; Group 3, 5xFAD + 4 J cm^−2^; Group 4, 5xFAD + 4 J cm^−2^‐BV2/mock‐exos; Group 5, 5xFAD + 4 J cm^−2^‐BV2/mmu‐miR‐7670‐3p‐mimic‐exos; Group 6, 5xFAD + 4 J cm^−2^‐BV2/mmu‐miR‐7670‐3p‐inhibitor‐exos. Data are presented as mean ± SD, n = 6 per group. ^*^p < 0.05, ^**^p < 0.01, ^***^p < 0.001, and ^****^p < 0.0001 versus the control group; *^#^p* < 0.05, *^##^p* < 0.01, *^###^p* < 0.001, and *^####^p* < 0.0001 versus the indicated group.

Dendritic spine dysfunction is a key feature of AD pathogenesis. Dendritic spines are specialized structures that directly relate to synaptic plasticity.^[^ ^30^ ^]^ In this study, we investigated the effect of exosomes derived from 4 J cm^−2^ 1070‐nm light‐treated BV2 cells on dendritic spines and synapses. Golgi staining and 3D reconstruction were used to track the dendritic morphology of stained neurons (**Figure** **11**a,d). Sholl analysis revealed that the number of intersections of dendritic arborizations was higher, and the dendritic morphology analysis indicated more intensive spine density in the 5xFAD mice treated with 4 J cm^−2^‐BV2/mmu‐miR‐7670‐3p‐mimic‐exos compared to those treated with 4 J cm^−2^‐BV2/mock‐exos (Figure 11a–e; Figure S5, Supporting Information). In contrast, 4 J cm^−2^‐BV2/mmu‐miR‐7670‐3p‐inhibitor‐exos resulted in a significant reduction in the number of intersections and dendritic spines in 5xFAD mice compared to the other groups. The data showed no significant differences in the 4 J cm^−2^‐BV2/mock‐exos group relative to data on the 4 J cm^−2^ 1070‐nm light group, although a slight increase was noted. Glutamate receptors and synaptic proteins in the cortex and HPC were also determined in the different groups (Figure 11f). The results showed that glutamate receptor 1 (GluR1), GluR2, N‐methyl‐D‐aspartic acid receptor 1 (NR1), NR2A, NR2B, postsynaptic density‐95 (PSD95), synapsin‐1, and the p‐CREB/CREB ratio significantly decreased in the 5xFAD mice from the control group (Figure 11g–n). In the group treated with 4 J cm^−2^‐BV2/mmu‐miR‐7670‐3p‐mimic‐exos, however, the number of glutamate receptors and synaptic proteins increased. The synapse protective effect was repressed by the treatment inhibitor. These results indicated that exosomal mmu‐miR‐7670‐3p derived from 4 J cm^−2^ 1070‐nm light‐treated microglia protected dendritic morphology and synaptic plasticity, thus mitigating cognitive function loss by activating memory‐related proteins.

### 2.13 Exosomes Derived from 1070‐nm Light‐Treated Microglia Alleviated ER Stress by Targeting ATF6 in 5xFAD Mice

**Figure 12:**
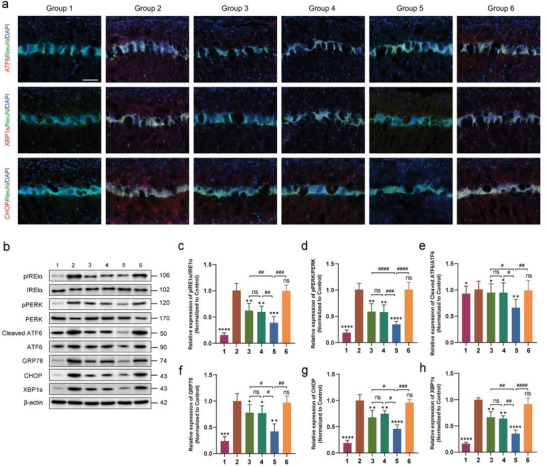
Exosomal mmu‐miR‐7670‐3p alleviated ER stress by targeting ATF6 in 5xFAD mice. a) Immunostaining for co‐localization of ATF6, XBP1s, and CHOP with NeuN in the cortex and CA1 in 5xFAD mice. Scale bar = 100 µm. b–h) Western blotting and quantitative analysis of IRElα, p‐IRElα, PERK, p‐PERK, ATF6, cleaved ATF6, GRP78, CHOP, and XBP1s in brain extracts of the cortex and HPC. Group 1, WT; Group 2, 5xFAD; Group 3, 5xFAD + 4 J cm^−2^; Group 4, 5xFAD + 4 J cm^−2^‐BV2/mock‐exos; Group 5, 5xFAD + 4 J cm^−2^‐BV2/mmu‐miR‐7670‐3p‐mimic‐exos; Group 6, 5xFAD + 4 J cm^−2^‐BV2/mmu‐miR‐7670‐3p‐inhibitor‐exos. Data are presented as mean ± SD, n = 6 per group. ^*^p < 0.05, ^**^p < 0.01, ^***^p < 0.001, and ^****^p < 0.0001 versus the control group; *^#^p* < 0.05, *^##^p* < 0.01, *^###^p* < 0.001, and *^####^p* < 0.0001 versus the indicated group.

In this study, we aimed to determine whether excessive ER stress was activated in the brain of 5xFAD mice. We conducted an analysis of relevant ER stress protein markers, including p‐IRE1α, inositol‐requiring enzyme 1 alpha (IRE1α), PKR‐like endoplasmic reticulum kinase (PERK), p‐PERK, ATF6, cleaved ATF6, C/EBP homologous protein (CHOP), glucose‐regulated protein 78 (GRP78), and XBP1s in the cortex and HPC via western blotting (**Figure** **12**b–h). Our results revealed that the levels of p‐IRE1α, p‐PERK, cleaved ATF6, CHOP, GRP78, and X‐box binding protein 1s (XBP1s) were significantly increased in the 5xFAD group compared to the control group. However, the levels of these proteins were significantly reduced in the mmu‐miR‐7670‐3p‐mimic‐BV2‐exos group. Based on the luciferase reporter assay results (Figure 6d), we hypothesized that exosomes derived from 4 J cm^−2^ 1070‐nm light‐treated microglia alleviated ER stress by downregulating ATF6 protein levels. The levels of ATF6 and cleaved ATF6 were noticeably reduced in the 5xFAD mice treated with mmu‐miR‐7670‐3p‐mimic‐BV2‐exos, leading to remission of ER stress. This alleviation of ER stress was also observed in the groups treated with 4 J cm^−2^ 1070‐nm light and mock‐BV2‐exos. However, the mmu‐miR‐7670‐3p‐inhibitor‐BV2‐exos treatment reversed this effect. Additionally, we found that ATF6, XBP1s, and CHOP were co‐localized with pyramidal neurons in the CA1 region (Figure 12a). The findings suggest that ATF6 is interconnected with the IRE1α‐XBP1 pathway and upregulated inflammatory mediators via the XBP1s/CHOP/NF‐κB pathway.^[^ ^27^, ^28^, ^29^ ^]^ In the CA1 region, we observed co‐localization of ATF6, XBP1s, and CHOP with pyramidal neurons, as shown in Figure 12a. This finding supports the hypothesis that exosomes derived from 4 J cm^−2^ 1070‐nm light‐treated microglia might alleviate ER stress by downregulating ATF6 expression.

## 3 Discussion

PBM refers to the therapeutic application of visible or NIR light for stimulating healing, alleviating pain and inflammation, and preventing tissue necrosis.^[^ ^8^, ^9^, ^10^, ^11^ ^]^ The prime mechanism of these effects of light is considered to be associated with cytochrome c oxidase (CCO), a mitochondrial protein complex that absorbs light in the range of 600–1000 nm.^[^ ^31^, ^32^ ^]^ The absorption of photons specifically stimulates an augmentation of electrons accessible for the reduction of molecular oxygen within the catalytic center of CCO. As a result, an elevation in the mitochondrial membrane potential (MMP) and levels of ATP, cyclic adenosine monophosphate (cAMP), and reactive oxygen species (ROS) is observed.^[^ ^33^ ^]^ These alterations signify an enhancement in mitochondrial function and have the potential to initiate cellular signaling pathways. It is known that NIR light above 980 nm cannot be absorbed by CCO and cannot stimulate the production of ROS to initiate the cellular effect.^[^ ^34^, ^35^ ^]^ However, in this study, we observed that low doses of NIR light at 1070 nm affected the morphological transformation of microglial cells, cell viability, and the release of inflammatory factors. There may be other potential mechanisms, in addition to CCO, through which NIR above 980 nm causes cellular effects. Recently, there have been suggestions that the infrared region of the spectrum can affect other chromospheres, even when it interacts with wavelengths above 900 nm. Ravera et al. discovered that the impact of 1064 nm light on the extrinsic mitochondrial membrane complex II and mitochondrial matrix enzymes was negligible; however, it exhibited a significant effect on the transmembrane mitochondrial complexes I, III, IV, and V.^[^ ^36^ ^]^ Yokomizo et al. observed that low‐power NIR‐II of 1064 nm and 1270 nm augmented nitric oxide bioavailability in endothelial cells and promoted cell migration via mitochondrial retrograde signaling.^[^ ^37^ ^]^ Another possible explanation is that water functions as the primary chromophore for infrared wavelengths above 900 nm within tissues and cells. Specifically, at 1070 nm, interactions between light and water at the nanoscale can result in physical alterations that impact ion channels on intracellular membranes, subsequently inducing cellular effects.^[^ ^38^, ^39^, ^40^, ^41^ ^]^

Microglia, as immune and inflammation‐regulating cells in the brain, play a more significant role in influencing the progression of Alzheimer's disease compared to oligodendrocytes and astrocytes, according to previous studies.^[^ ^42^, ^43^, ^44^ ^]^ Therefore, we selected microglia for further NIR photobiomodulation. Previous studies demonstrated the modulation of M1/M2 polarization of microglia under NIR treatment.^[^ ^16^, ^45^, ^46^, ^47^ ^]^ Stepanov et al. observed that red/NIR light significantly increased the percentage of M2 macrophages secreting VEGF and promoting endothelial cell proliferation.^[^ ^45^ ^]^ Leden et al. showed that low‐energy density 808‐nm light altered M1/M2 polarization and reduced the release of pro‐inflammatory factors.^[^ ^46^ ^]^ Similarly, PBM at 808 nm after ischemic stroke of rats transformed M1 microglia into anti‐inflammatory M2 microglia, thus improving the neuronal microenvironment by altering the inflammatory status.^[^ ^47^ ^]^ In a previous study, a decrease in the M1 phenotype microglia was observed in the cortex of AD mice after 1070‐nm light treatment.^[^ ^16^ ^]^ In the current study, we found that 1070‐nm light pulsed at 10 Hz activated BV2 cells in vitro and microglia in vivo by increasing M1/M2 polarization. According to Arndt and Schulz's law, PBM effects are dependent on the tissue, wavelength, and energy dosage. Higher doses of energy result in inhibition, while low to moderate energy fluences induce activity.^[^ ^48^ ^]^ In this study, we used energy dose gradients of 1070‐nm light for irradiating BV2 cells. We observed a significant increase in the M2 phenotype of BV2 cells when treated with a dose of 4 J cm^−2^ rather than 2 J cm^−2^, 6 J cm^−2^, or 8 J cm^−2^. This finding is consistent with Leden et al., who observed a significant increase in the M2 phenotype of microglia treated with 4 J cm^−2^ of 808‐nm light compared to 0.2 J cm^−2^ and 10 J cm^−2^.^[^ ^46^ ^]^ Moreover, as the energy fluence increases, the cell viability of microglia treated with 6 J cm^−2^ or 8 J cm^−2^ decreases accordingly compared to relatively low energy fluences, such as 2 J cm^−2^ or 4 J cm^−2^. Therefore, we selected an optimal irradiation parameter of 4 J cm^−2^ for cell irradiation for further exploration in this study.

Exosomes are implicated in the pathogenesis of AD, but their exact role is still not fully understood. Some researchers believe that activated microglia secrete exosomes that spread Aβ and p‐tau, thereby contributing to the neurotoxicity observed in AD.^[^ ^49^, ^50^, ^51^ ^]^ However, other studies suggest that exosome production alleviates the pathological phenotype of AD by delivering neurotrophic factors, restoring synaptic function, or transporting Aβ to microglia for clearance.^[^ ^52^, ^53^, ^54^, ^55^ ^]^ Microglia‐derived exosomes have been shown to mediate neuronal survival, neurite outgrowth, and neuroinflammatory responses through enzymes, chaperones, membrane receptors, and miRNAs.^[^ ^56^ ^]^ However, the effects of exosomes secreted by NIR‐modulated M2 microglia on the pathological process of AD remain unknown. As non‐coding RNAs, miRNAs have been extensively studied for their role in various biological activities such as cell proliferation, differentiation, migration, and disease initiation and progression.^[^ ^57^, ^58^, ^59^ ^]^ Exosomal miRNAs are considered key functional elements between cell interactions compared to other exosomal cargos.^[^ ^60^, ^61^ ^]^ In this study, we demonstrated that 1070‐nm light enhanced M2 microglia polarization and induced the secretion of exosomes containing miRNAs that play a critical role in tube morphogenesis, synapse formation, and dendrite development. Moreover, exosomal miRNAs derived from 1070‐nm light modulated M2‐microglia also led to increased cell viability and reduced expression of inflammatory cytokines such as IL‐1β, TNF‐α, and IL‐6, as well as Aβ peptides associated with AD pathology.

In AD, ER stress is critical in determining disease progression, as it activates the UPR to restore ER homeostasis.^[^ ^62^ ^]^ However, excessive stress can impair mitochondrial function, promote inflammation, and trigger programmed cell death of neurons.^[^ ^26^, ^62^, ^63^ ^]^ The core proteins that initiate this evolutionarily conserved response in mammalian cells are ATF6, PERK, and IRE‐1α.^[^ ^64^ ^]^ We found that miR‐7670‐3p, which targets ATF6, was upregulated in exosomes secreted by BV2 cells and brain tissue after 1070‐nm light irradiation. Under normal conditions, p90ATF6 is constitutively expressed in the perinuclear region of the cell. ER stress induces the conversion of p90ATF6 to p50ATF6, which is translocated into the nucleus and directs the expression of genes involved in protein folding and ER‐associated degradation .^[^ ^65^, ^66^ ^]^ The evidence shows that cleaved ATF6 is intimately interconnected with the IRE1α‐XBP1 pathway. ATF6 partially regulates XBP1 expression.^[^ ^27^ ^]^ Furthermore, previous studies suggest that the activation of ATF6 can also trigger the expression of inflammatory mediators via the XBP1/CHOP/NF‐κB pathways.^[^ ^27^, ^28^, ^29^ ^]^ As a downstream effector of the p38MAPK pathway, NF‐κB and its downstream signaling molecules play important roles in triggering the inflammatory response. NF‐κB enhances the activation of the NLRP3 inflammasome, which prompts the release of mature cytokines IL‐1β and upregulates caspase‐1‐dependent pyroptosis, leading to an inflammatory cascade .^[^ ^67^, ^68^ ^]^ In our study, we observed high levels of ER stress in 5xFAD mice. ER stress and neuroinflammation was attenuated after treatment with exosomes derived from 4 J cm^−2^ light‐treated microglia, an miR‐7670 mimic, or 1070‐nm light. Therefore, our results indicated that 1070‐nm light pulsed at 10 Hz might suppress chronic neuroinflammation and prompt neuronal survival by mediating ER stress. Inflammation‐promoting chronic stress may create a vicious cycle by increasing Aβ accumulation in a positive feedback loop.^[^ ^69^ ^]^ Lv et al. found that suppressing inflammation via AMPKα1/IκB/NF‐κB signaling contributed to reducing amyloid plaque deposition in APP/PS1 mice.^[^ ^70^ ^]^ Similarly, we found that Aβ accumulation in the cortex and HPC of 5xFAD mice decreased after attenuating the neuroinflammatory response by reducing ER stress.

In this study, we observed an upregulation in the expression of postsynaptic proteins, accompanied by a decrease in ER stress, Aβ accumulation, and neuroinflammation in 5xFAD mice after therapy. The loss of synaptic connections is considered the primary anatomical correlate of cognitive decline in AD. Aβ oligomers have been found to reduce synaptic plasticity and inhibit long‐term potentiation,^[^ ^71^ ^]^ while high levels of ER stress can lead to synaptic deficits through the PERK/eIF2α pathway. This pathway activates PERK and induces eIF2a phosphorylation, which impaired synaptic plasticity and memory performance in AD.^[^ ^72^, ^73^, ^74^ ^]^ Thangwong et al. reported that suppressing ER stress increased the expression of synaptic markers and improved cognitive performance in a rat model of chronic cerebral hypoperfusion.^[^ ^75^ ^]^ Neuroinflammation can also affect synaptic morphology and promote neurodegeneration, as shown in previous studies.^[^ ^74^, ^76^ ^]^ Brigas et al. demonstrated that inflammatory cytokines triggered the onset of cognitive and synaptic deficits in the early stages of AD.^[^ ^77^ ^]^ Lonnemann et al. also found that an NLRP3 inflammasome inhibitor rescued synaptic plasticity in APP/PS1 mice.^[^ ^78^ ^]^ Our findings suggest that 1070‐nm light reduced ER stress levels in neurons, decreasing the generation and accumulation of Aβ peptides and reducing synaptic deficits. Another possible explanation for the effects of 1070‐nm light on synaptic plasticity is that it suppressed neuroinflammation by inhibiting ER stress, reducing the negative impact of the inflammatory microenvironment on the synapse.

## 4 Experimental Section

The current study revealed that 1070‐nm light at 10 Hz increased M2‐like microglia and decreased M1‐like microglia both in vivo and in vitro. Exosomes derived from 1070‐nm light activated M2‐like microglia contained anti‐inflammatory miR‐7670‐3p that targeted ATF6 to attenuate ER stress in neurons of the cortex and HPC in 5xFAD mice, leading to the suppression of neuroinflammation, increased synaptic plasticity, and improvements in learning and memory. Furthermore, reducing neuroinflammation in neurons decreased the Aβ burden in response, as there is a positive correlation between the neuroinflammatory response and Aβ burden. One potential limitation of this research is the use of an animal model, which may not entirely reflect the pathological condition of AD in humans. Nonetheless, this work provides valuable insights into the therapeutic effects and mechanisms of 1070‐nm light in the treatment of AD.

### Cell Culture

Murine microglial BV2 cells were maintained in DMEM/F12 medium (Gibco, USA) supplemented with 10% fetal bovine serum (Gibco, USA), and 1% penicillin/streptomycin at 37 °C in a humidified incubator with 5% CO_2_. Cells were passaged at 80–90% confluence. Before treatment, cells were plated and allowed to grow for 24 h. Mouse neuroblastoma N2a cells stably expressing the Swedish mutant gene APP695 (N2a/APP695swe) were donated by Professor Huaxi Xu (Xiamen University, Xiamen, China) and Professor Yingjun Zhao (Xiamen University, Xiamen, China). Cells were cultured in MEM (Gibco, USA) supplemented with 5% fetal bovine serum (Gibco, USA).

### Treatment of Cells with 1070‐nm Light

For in vitro experiments, all groups of cells were treated with various chemicals and/or irradiated with a 1070‐nm light emitting diode (LED) array. The average power density was 12 mW cm^−2^. The transmittance of the plastic cover of the culture dish was ≈86.2%. LED irradiation was used at a frequency of 10 Hz (duty cycle: 50%) with cooling fans to prevent thermal effects.

For 1070‐nm light treatment, cells in the culture dish were placed on the platform of the 1070‐nm light device and received treatment for 7, 14, 21, or 28 min with penetrating fluences of 2 J cm^−2^, 4 J cm^−2^, 6 J cm^−2^ or 8 J cm^−2^, at 10 Hz, respectively. The entire experiment was performed at room temperature. To minimize light interference, the cells were kept in either completely dark or very dim light during every experiment, except when light irradiation was applied. Then, cells were maintained at 37 °C in a dark incubator containing 5% CO_2_ before harvesting cells at 4 h for co‐culture.

### Co‐Culture of N2a/APP695swe Cells with BV2 Cells

BV2 cells were treated with LPS (100 ng mL^−1^) for 4 h or 1070‐nm light exposure. In the rescue experiment, the N‐SMase inhibitor GW4869 (20 µM) (Sigma‐Aldrich, USA) was added to the culture medium 30 min before 1070‐nm light treatment. Co‐culturing was performed with 0.4‐mm porous cell culture inserts (BD Falcon, USA). In total, 2×10^4^ N2a/APP695swe cells were seeded into the lower chambers of 24‐well plates (BD Falcon, USA). Twenty‐four hours later, ≈6×10^3^ BV2 cells were seeded into the upper chambers. For exosome treatment, exosomes were added to the lower chambers at 1 µg of exosomes per 5×10^4^ recipient cells without seeding BV2 cells in the upper chambers. Co‐cultures were maintained in the incubator for 24 h, and then cell medium and N2a and APP695swe cells were collected for further analysis (Figure 3a).

### Isolation of BV2‐exos

Twenty‐four hours after 1070‐nm light treatment, the cell medium of BV2 cells was centrifuged at 3000 g for 15 min to remove cell debris. Afterward, the supernatant was collected and mixed with an appropriate volume of ExoQuick TC (System Biosciences, USA) overnight at 4 °C. In accordance with the manufacturer's instructions, the mixture was centrifuged repeatedly, and the supernatant was discarded to remove residual ExoQuick. Exosome pellets were dissolved in 500 µL of 1×PBS, and bicinchoninic acid was used to determine the protein concentrations.

### Labeling and Tracking of Exosomes

For tracking the exosomes, the red fluorescent dye PKH26 (Sigma‐Aldrich, USA) was applied to freshly harvested exosomes. In brief, 1.5 mg of exosomes were incubated for 10 min with a diluent C solution and PKH26 dye, followed by centrifugation through Amicon ultra‐0.5 mL filters (Millipore, USA). Exosomes were washed twice with PBS and resuspended in 500 µL of PBS. The PKH26 dye‐labeled exosomes were immediately added to the medium of N2a/APP695swe cells or intranasally administered to animals.

### Neurite Outgrowth and Dendritic Development Assay

Evaluation of neurite growth in N2a/APP695swe cells was modified from a previously described method.^[^ ^79^ ^]^ The number of branch points, the diameter of the first section from the soma, the longest neurite length, and the total length of neurites in each cell were measured by using the software ImageJ. Fifty neurons from three independent experiments were used for statistical analysis.

### RNA Interference

Mimics and inhibitors of mmu‐miR‐7670‐3p were synthesized by GenePharma (Suzhou, China). Sequences of synthetic miRNA mimics or inhibitors were listed in Tables S3 and S4 (Supporting Information). Lentivirus vectors expressing mmu‐miR‐7670‐3p and repressing mmu‐miR‐7670‐3p were constructed and generated by Genechem (Shanghai, China). For exosome transfection, mimics or inhibitor of mmu‐miR‐7670‐3p were loaded into exosomes using an Exo‐Fect exosome transfection kit (System Biosciences, USA).

### Intranasal Administration of Exosomes

Before administration of exosomes, mice received 10 µL per nostril of hyaluronidase (100 U, H3506; Sigma‐Aldrich, USA) in PBS to enhance mucous membrane permeability. Intranasal administration of exosomes or PBS was conducted by gently holding the ventral side of the mouse up with its head facing downward. In a sterile PBS solution, 200 g mL^−1^ of BV2‐exos were suspended and stored at −80 °C. Approximately 2.8 × 10^9^ exosomes in a total volume of 20 µL were dispensed into the two sides of the nostril in 5 µL spurts separated by 5 min. Intranasal administration was performed every second day, and each animal received 26 doses over 2 months before the behavioral test.

### Animals

Four‐month‐old male 5xFAD transgenic mice and WT littermates were obtained from JOINN Laboratories (Peking, China). Mice were divided into six groups: negative control group (WT), sham treatment (5xFAD), 4 J cm^−2^ 1070‐nm light irradiation (5xFAD + 4 J/cm^2^), 4 J cm^−2^‐BV2/mock‐exos treatment (5xFAD + 4 J cm^−2^‐BV2/mock‐exos), 4 J cm^−2^‐BV2/mmu‐miR‐7670‐3p‐mimic‐exos treatment (5xFAD + 4 J cm^−2^‐BV2/mmu‐miR‐7670‐3p‐mimic‐exos), and 4 J cm^−2^‐BV2/mmu‐miR‐7670‐3p‐inhibitor‐exos treatment (5xFAD + 4 J cm^−2^‐BV2/mmu‐miR‐7670‐3p‐inhibitor‐exos). The 5xFAD group and negative control group represented mice without treatment and normal mice, respectively. The light irradiation procedure on animals was performed in vivo using a modified near‐infrared LED array from Tao et al.^[^ ^16^ ^]^ (Figure S6, Supporting Information). Briefly, mice in the 5xFAD + 4 J cm^2^ group received penetrating irradiation of 4 J cm^−2^ at 10 Hz in the 1070‐nm light device every second day for 2 months before behavioral tests. During treatments, mice were allowed to explore and rest in the chamber freely. The mice were kept on a light‐dark cycle of 12/12 h with sufficient food and water. Animal care and experimental protocols were approved by the Fudan University Ethical Committee of Animal Experiments. After behavioral tests in the last 7 days (Figure 8a, Supporting Information), mice were humanely sacrificed. Brains were dissected, frozen on dry ice, and stored at −80 °C until further processing.

### Tissue Preparation

After anesthesia with isoflurane, mice were transcardially perfused with 0.9% saline. Afterward, the brains were removed from the skull and separated into two hemispheres, followed by postfixing overnight in 4% paraformaldehyde or fresh‐frozen on ice. Brains were removed from the hemispheres and homogenized for further processing. For cryoprotection, the brains were immersed in a 10% sucrose solution for one night, then in a 30% sucrose solution for at least one night. The brains were embedded in optimum cutting temperature compound (Tissue‐Tek, USA) and mounted on a freezing microtome (Leica CM1900, Germany). The coronal sections were made with a microtome at a 30–70‐µm thickness.

### Golgi Staining and Dendritic Development Assay

Briefly, the brains of 6‐month‐old mice were dissected and surface blood was removed. For 14 days, the brains were immersed in FD solutions A and B (FD Neurotechnologies, USA) and then for 7 days in FD solution C at room temperature in the dark. After that, brains were sectioned into 100‐µm thicknesses and mounted onto gelatin‐coated slides with the FD C solution before drying at room temperature. Staining was carried out according to the manufacturer's instructions. Pyramidal neurons located in the CA1 region of the HPC was captured with a Nikon C2+ Si confocal microscope (Nikon, Japan) with Z‐stack series. Amira software (Thermo Fisher Scientific, USA) was used to reconstruct individual Z‐stack images into 3D and measure the density of spines. Morphological alterations of six random neurons at the HPC region of each animal from each group were manually measured using ImageJ software. The number of intersections, distance from the soma, and density of the spines were measured and analyzed.

### Immunofluorescence Staining

Cells were washed in PBS and fixed in 4% paraformaldehyde for 15 min before being washed twice with PBS for 5 min each time. Following incubation in blocking buffer (0.1% Triton X‐100 and 1% BSA in PBS) for 30 min, cells or brain slices were incubated in primary antibody overnight at 4 °C. The antibodies were listed in Table S1 (Supporting Information). Cells or brain slices were then washed three times with PBS for 10 min each time and subsequently reacted with Alexa Fluor 488 (1:1000, ab150081, Abcam), Alexa Flour 594 (1:1000, ab150120, Abcam), or Alexa Fluor 647 (1:1000, ab150083, Abcam) secondary antibodies for 1 h at room temperature, and washed three times with PBS. Coverslips were mounted on slides using Vecta‐Shield anti‐fade mounting medium (Vector Laboratories, USA). A BX43 fluorescence microscope (Olympus, Japan) or Pannoramic SCAN (3DHISTECH, Hungary) was used for imaging. Qualitative assessments were measured using ImageJ software.

### Flow Cytometry

After treatment for 4 h, BV2 cells were centrifuged at 500 g for 5 min at 4 °C to harvest and then washed twice with a 0.5% BSA‐PBS solution. Then, cell pellets were suspended in 1 mL of 0.5% BSA‐PBS and counted using a cell counter. Cells were preincubated with mouse Fc for blocking (BD Biosciences, USA) for 30 min. Then the cell suspension was stained with anti‐CD86 (105 005, Biolegend) or anti‐CD206 (141 703, Biolegend) and loaded into the flow cytometer for detection. Data were analyzed using FlowJo (FlowJo, LLC).

### Western Blotting

For western blotting, following determination of protein concentration and denaturation, a Trans‐Blot apparatus (Bio‐Rad) was used to electroblot proteins onto an Immobilon‐P membrane after separation by SDS‐PAGE. After blocking in PBS containing 5% nonfat milk for 1 h at room temperature, membranes were cleaned in distilled water. The membranes were then incubated with antibodies overnight at 4 °C, which were listed in Table S1 (Supporting Information). Then the blots were incubated with horseradish peroxidase‐conjugated secondary antibody (1:15000) for 2 h at room temperature. The bands were visualized with the aid of a GelDoc XR system (Bio‐Rad, USA). Using ImageJ software, protein levels were determined by analyzing the protein bands and comparing their intensity ratios to control bands.

### Enzyme‐Linked Immunosorbent Assay (ELISA)

The concentration of Aβ_40_ and Aβ_42_ in the N2a/APP695swe cells, cell culture medium, and brain tissue were detected by ELISA kits (Invitrogen, Camarillo, CA). Inflammatory cytokines, including TNF‐α, IL‐1β, and IL‐6 were determined using ELISA kits (Signalway Antibody, USA) according to the manufacturer's instructions. After centrifugation, the supernatants or brain homogenates were diluted 1:10. For intracellular Aβ analysis, cells in a protease inhibitor‐containing protein lysis buffer (Beyotime, China) and determined the protein concentration with a BCA protein assay. Detergent‐insoluble Aβ was detected by extracting pellets in 5 M guanidine HCl buffer, followed by a 1:20 dilution in lysis buffer. Based on the optical density value measured at 450 nm, the results were converted to picograms per milligram of protein.

### Quantitative RT‐PCR (qRT‐PCR) Analysis

Samples containing BV2 cells, N2a/APP695swe cells, exosomes, or brain hemispheres were thawed on ice. Following the manufacturer's instructions, total RNA was extracted by using the Trizol reagent (Clontech Laboratories, USA). A Mir‐X miRNA First‐Strand synthesis kit for miRNAs (Clontech Laboratories, USA) and PrimeScript RT master mix for genes (Clontech Laboratories, USA) were used for reverse transcription. Real‐time PCR was carried out on a ViiA7 Real‐Time PCR system (Applied Biosystems, CA, USA) by using SYBR Green PCR master mix (Applied TaKaRa, Shiga, Japan). The sequences of all indicated primers were listed in Table S2.

### Behavioral Tests

*Morris water maze test*: The Morris water maze test was performed as previously described.^[^ ^16^ ^]^ Mice were placed into a water tank with water at 22–24 °C, and a hidden platform was located at a 1.5 cm height under the water's surface in the center of the fourth quadrant. The mice underwent four trials with four starting positions every day before the final test. During each trial, the time to find the platform was recorded as the escape latency. After training for 5 days, the platform was removed, and each mouse was allowed to swim for 60 s in a random quadrant. The paths of the mice were recorded by a digital camera. The number of platform location crossings and time spent in the target quadrant were analyzed.

### Novel Object Recognition Test

The mice were pre‐exposed for 5 min to two identical objects in an open field chamber. Two hours after pre‐exposure, mice were returned to the chamber and exposed to familiar and novel objects for 5 min. Each group of mice was exposed to a specific object only once across different test sessions, and novel object placement was counterbalanced. An analysis was conducted on the retention rate for novel objects.

### Y‐Maze

In the maze, mice were allowed to explore for five minutes while the number and sequence of arm choices of the maze were recorded. The time spent by mice in the start arm, center zone, novel arm, and other arm was analyzed.

### Open Field Test

Test mice were positioned in the center of a circular open field chamber (55 cm diameter, 30 cm high). A circle with a 30‐cm radius (2827 cm^2^) was defined as the center zone. The average speed and time spent in the center zone were analyzed. A digital camera was used to record all animal behaviors.

### Statistical Analysis

GraphPad Prism version 8.0 was used to analyze the data. Two‐tailed unpaired *t* tests were used to analyze normally distributed data with two groups. In the case of normally distributed data with three or more groups, one‐way ANOVA was used to test comparisons. Non‐normally distributed data were analyzed using Mann‐Whitney tests. Statistical differences for all tests were considered significant at *p* < 0.05.

## Conflict of Interest

The authors declare no conflict of interest.

## Author's Contributions

C.C., Y.B., L.X. contributed equally to this work. C.C. conceived and designed the research. C.C., Y.G., Y.B., C.J., and S.T. performed the experiments. Images and data were processed by C.C., Y.G., and S.T. The manuscript was written by C.C., Y.G., and S.T. J.Z., L.C., and Y.M. supervised the project.

## Supporting information

Supporting Information

## Data Availability

The data that support the findings of this study are available from the corresponding author upon reasonable request.
