# Correction to “Exosomes Derived from M2 Microglial Cells Modulated by 1070‐nm Light Improve Cognition in an Alzheimer's Disease Mouse Model”

**DOI:** 10.1002/advs.202519668

**Published:** 2026-01-15

Chen C, Bao Y, Xing L, Jiang C, Guo Y, Tong S, Zhang J, Chen L, Mao Y. Exosomes Derived from M2 Microglial Cells Modulated by 1070‐nm Light Improve Cognition in an Alzheimer's Disease Mouse Model. Adv Sci (Weinh). 2023 Nov;10(32):e2304025. doi: 10.1002/advs.202304025

Upon a recent and careful review of our published article, we have identified two errors that require correction.

1. **Typographical Error in the Experimental Section**: In the subsection “Intranasal Administration of Exosomes,” we found a typographical error in the concentration unit. The text currently reads: “In a sterile PBS solution, 200 g mL‐1 of BV2‐exos were suspended and stored at −80 °C.” The correct unit should be “µg mL‐1.” This was an unintentional mistake during the final preparation of the manuscript. The corrected sentence should read: “In a sterile PBS solution, 200 µg mL‐1 of BV2‐exos were suspended and stored at −80 °C.”
2. **Image Misplacement and Overlap in Figure 1b**: We discovered an error in the assembly of Figure 1b. Due to an oversight, the immunofluorescence panel for the

**6 J cm^−^^2^ group (stained with CD206 + IBA1)** was inadvertently replaced with the image from the **2 J cm^−^^2^ group (stained with CD86 + IBA1)**. This misplacement also resulted in a partial overlap of two adjacent image panels. To clearly illustrate the issue, original Figure 1b, in which the misplaced image is highlighted with a red frame and the overlapping areas are marked with yellow frames.

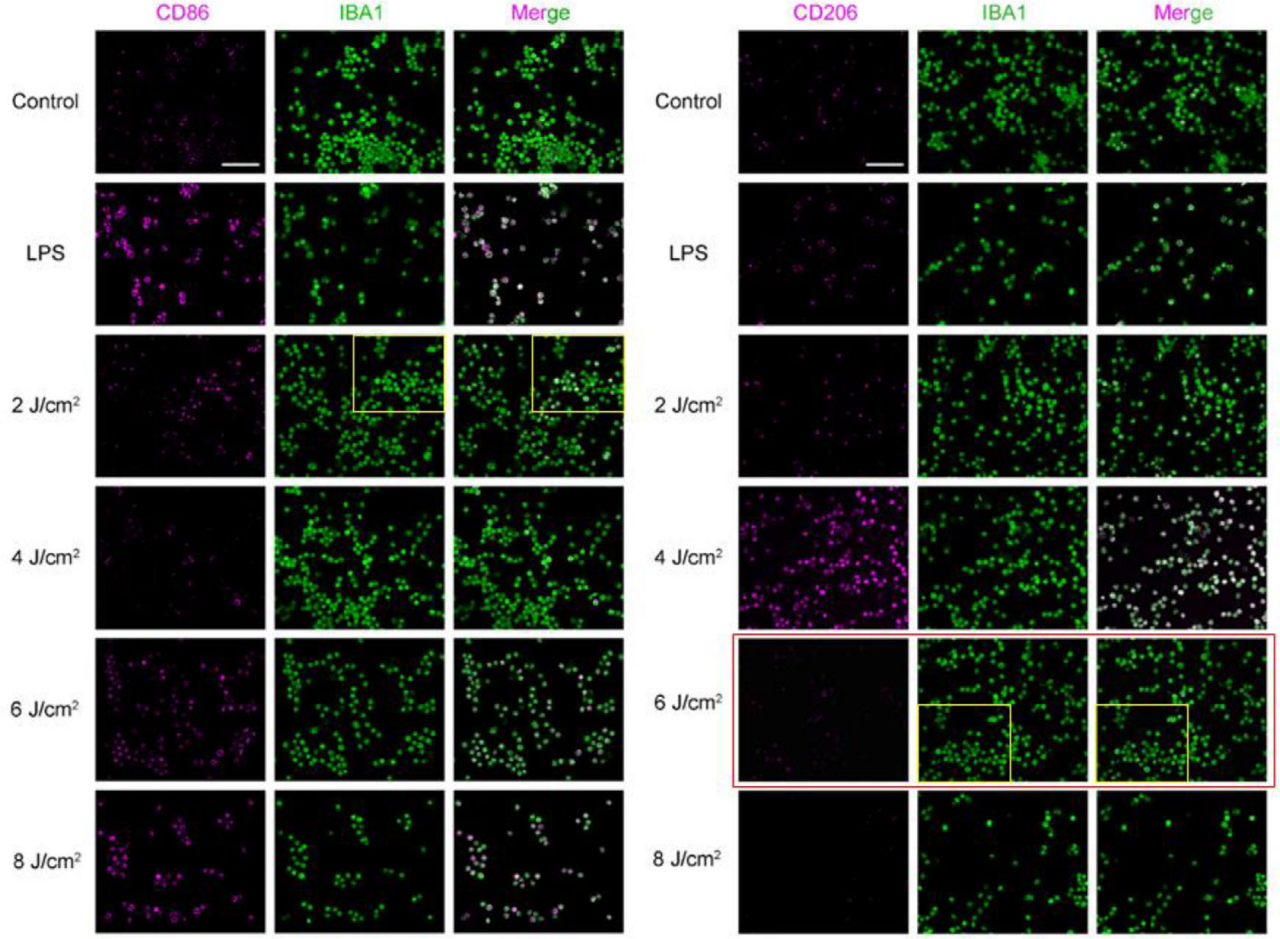

The corrected version of Figure 1b is shown below. It is crucial to note that this figure assembly error did not impact the experiment itself or the subsequent quantitative analysis.

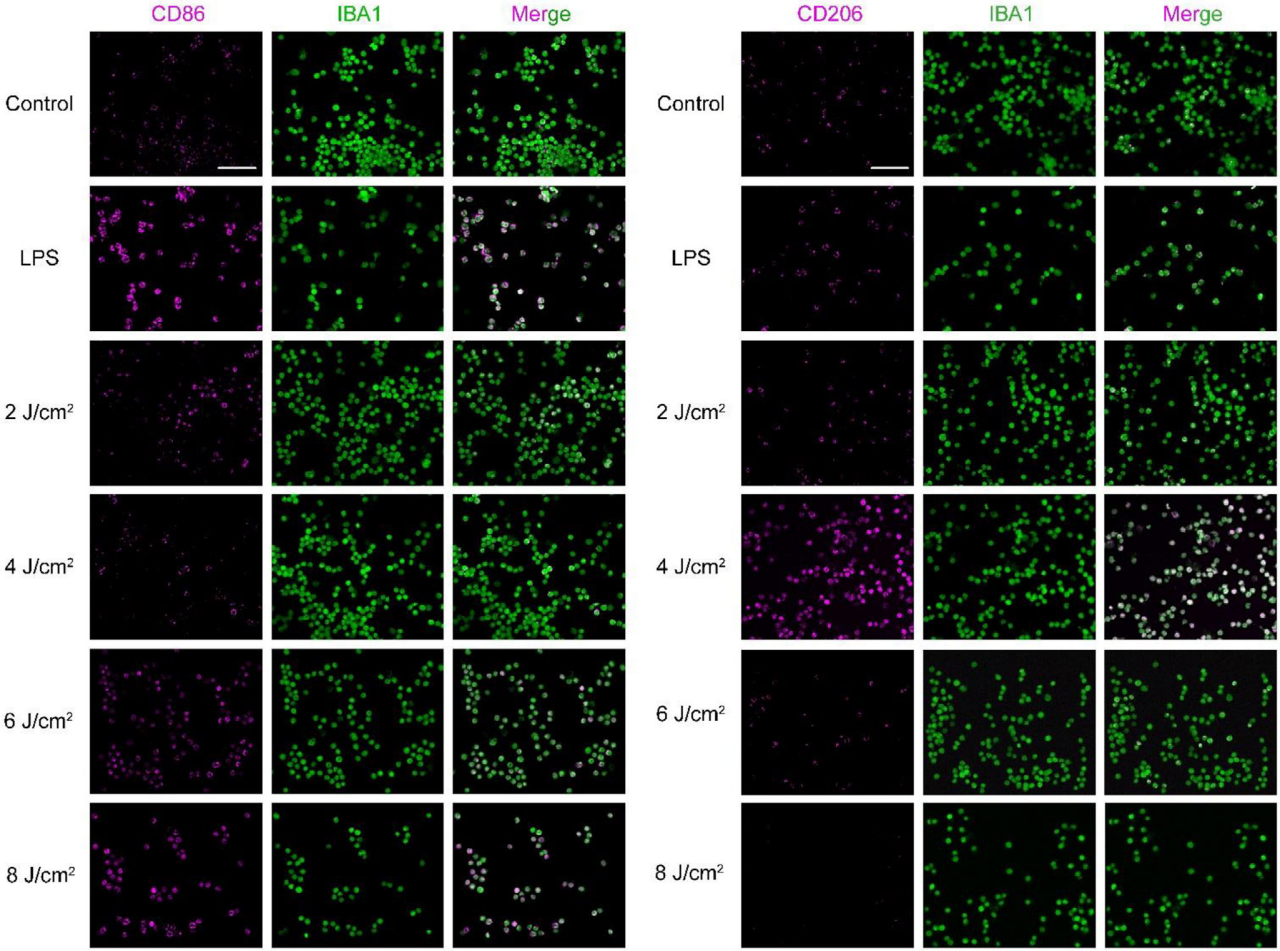

We apologize for this error.

