# Supplementary material for: Exosomes Derived from M2 Microglial Cells Modulated by 1070‐nm Light Improve Cognition in an Alzheimer's Disease Mouse Model
Source: Adv Sci (Weinh). 2023 Sep 13;10(32):2304025. doi: 10.1002/advs.202304025 (PMC10646245; doi:10.1002/advs.202304025)
Supplement: Supplementary file 1 — Supporting Information [file ADVS-10-2304025-s001.pdf]

ADVANCED SCIENCE
Open Access

Supporting Information

for *Adv. Sci.*, DOI 10.1002/advs.202304025

Exosomes Derived from M2 Microglial Cells Modulated by 1070-nm Light Improve Cognition in an Alzheimer's Disease Mouse Model

Chengwei Chen, Yuting Bao, Lu Xing, Chengyong Jiang, Yu Guo, Shuangmei Tong, Jiayi Zhang, Liang Chen* and Ying Mao**

Supporting information

Exosomes Derived from M2 Microglia Cells Modulated by 1070-nm Light Improve Cognition in Alzheimer's Disease Mouse Model

Chengwei Chen[#], Yuting Bao[#], Lu Xing[#], Chengyong Jiang, Yu Guo, Shuangmei Tong, Jiayi Zhang[*], Liang Chen[*], Ying Mao[*]

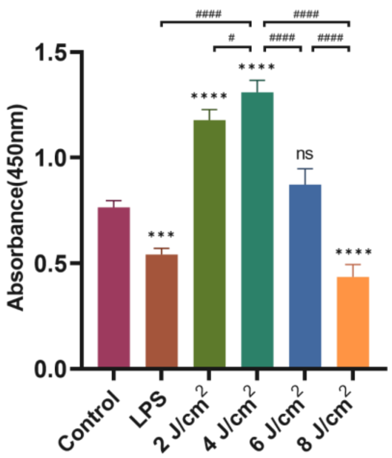

Figure S1. Cell proliferation of BV2 microglial cells in response to different treatment. CCK8 viability assay for BV2 cells at 24 hours after different treatment. The results were based on three repeated experiments. Data are presented as mean ± SD. $^{*}p < 0.05$, $^{**}p < 0.01$, $^{***}p < 0.001$ and $^{****}p < 0.0001$ versus the control group; $^{\#}p < 0.05$, $^{\#\#}p < 0.01$, $^{\#\#\#}p < 0.001$ and $^{\#\#\#\#}p < 0.0001$ versus the indicated group.

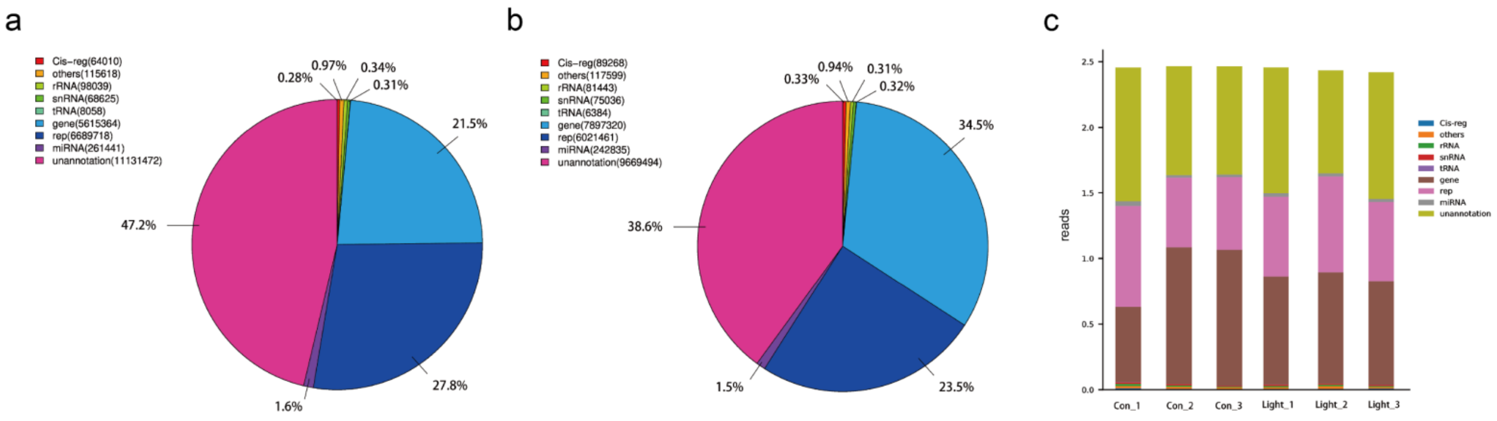

Figure S2. Classification of reads of small RNAs for BV2 cells derived exosome. a. pie chart of reads categorized annotation for the BV2-exos from control group. b. pie chart of reads categorized annotation for the BV2-exos from 4 J/cm^2 1070-nm light treated group. c. the bar chart shows the number of reads for various types of small RNAs.

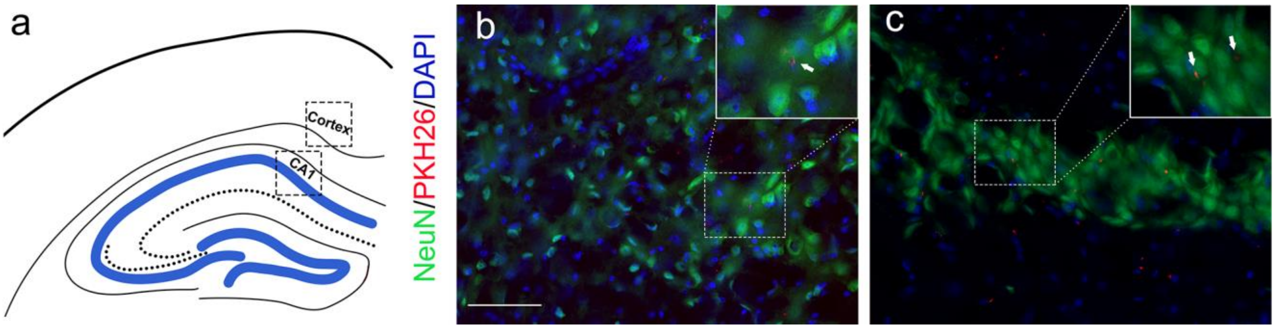

Figure S3. Uptake of BV2-exos by neurons in the pathological areas of 5xFAD mice 24 h after intranasal administration. a. Schematic shows the distribution of BV2-exos to the cortex and CA1 of HPC. b. NeuN labeling of neurons shows PKH26 labeled BV2-exos homing to the cortex of 5xFAD mice. c. NeuN labeling of neurons in the HPC shows PKH26 labeled BV2-exos colocalized with neurons in CA1. Arrowheads show the uptake of PKH26-labed exosomes by neuron. Scale bar = 50 μm.

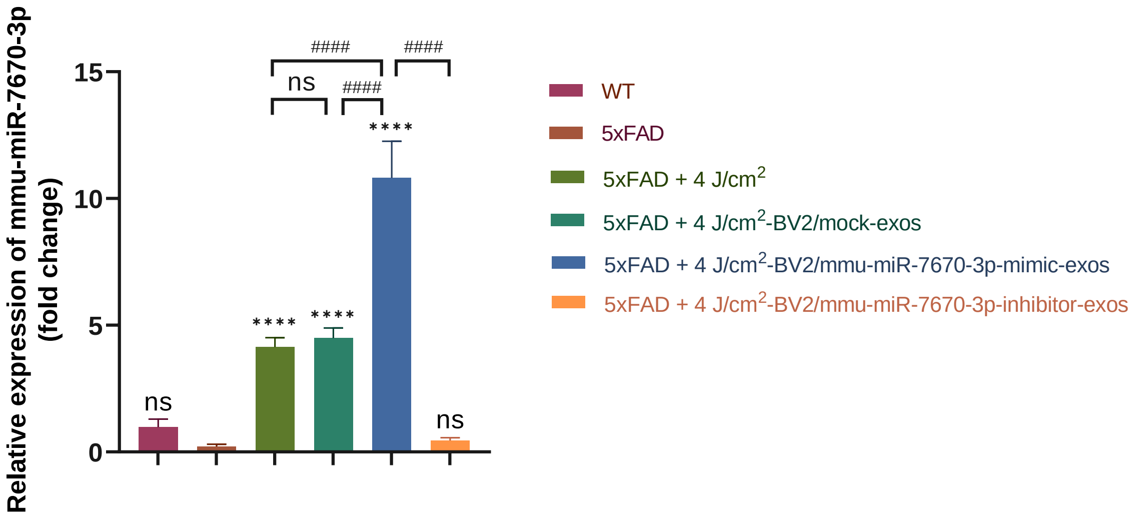

Figure S4. mmu-miR-7670-3p is highly expressed in 5xFAD treated with 4 J/cm^2 light, 4 J/cm^2-BV2/mock-exos or 4 J/cm^2-BV2/mmu-miR-7670-3p-mimic-exos. qRT-PCR analysis detecting the expression level of mmu-miR-7670-3p in brain tissue of mice with different treatment.

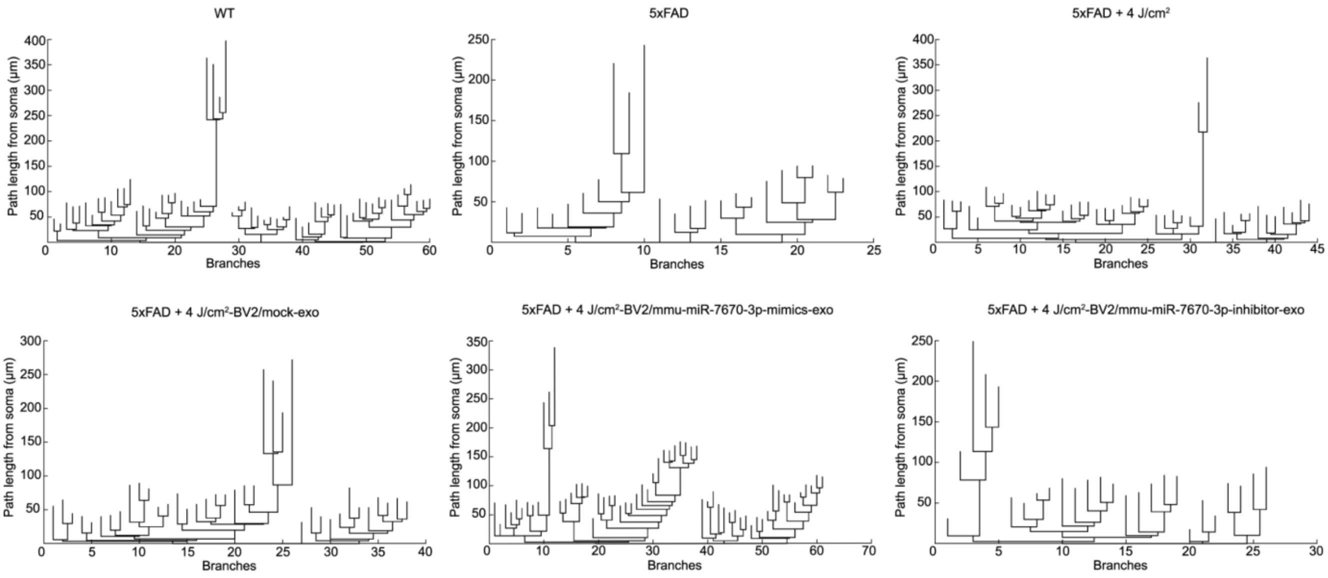

Figure S5. Corresponding dendrograms for typical dendrites from pyramidal neurons within CA1 in mice with different treatment. Both the path length from pyramidal neuronal soma and branches of individual dendritic segments are illustrated by dendrograms.

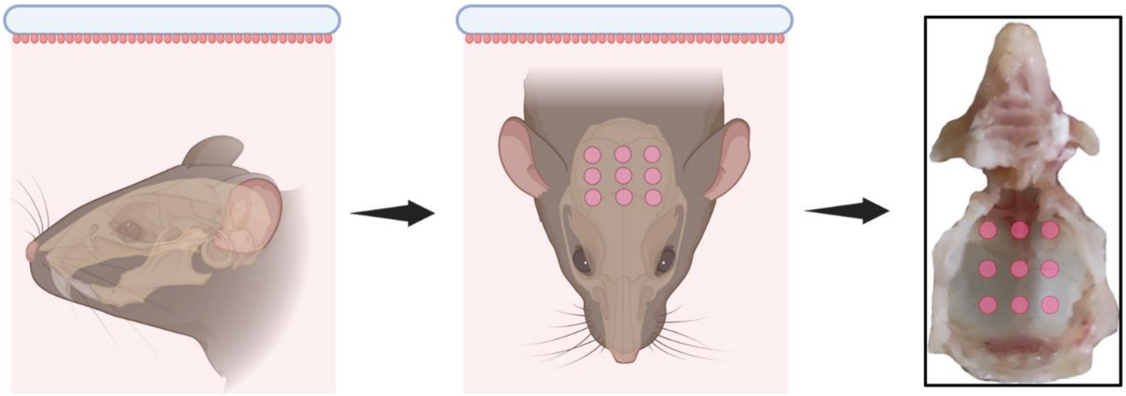

Anatomical structure	Power density (mW/cm^2)	Transmittance (%)
Scalp	9.86 ± 0.42	7.92 ± 1.64
Scalp and skull	5.09 ± 0.23	4.15 ± 0.48

Figure S6. Measurement of the parameters of the 1070-nm light irradiation apparatus. The 1070-nm light irradiation apparatus, composed of a light-emitting diode (LED) array, was modified from Tao et al.[1]. The LED array had an average power of 75 W and a power density of 125 mW/cm^2. The power meter was used to measure the power density and transmittance of 1070-nm light. The red dot on the graph indicates the measured location, which corresponds to the positions of cranial outer and inner tables (image created with Biorender.com).

Table S1. Primary antibodies used in this study.

Antibody	Dilution	Cat. No.	Source
CD206	1:1000 for WB<br>1:200 for IHC	ab64693	Abcam (Cambridge, UK)
CD86	1:1000 for WB<br>1:100 for IHC	ab119857	Abcam (Cambridge, UK)
Iba1	1:100 for IHC	ab283346	Abcam (Cambridge, UK)
βIII-tubulin	1:500 for IHC	ab52623	Abcam (Cambridge, UK)
NeuN	1:500 for IHC	ABN78	Millipore (MA, USA)
NLRP3	1:1000 for WB<br>1:200 for IHC	768319	Invitrogen (Camarillo, CA)
ASC	1:1000 for WB	AG-25B-0006	Adipogen Life Sciences
Cleaved Caspase-1	1:1000 for WB	AG-20B-0042	Adipogen Life Sciences
Cleaved IL-1β	1:1000 for WB	ab9722	Abcam (Cambridge, UK)
GSDMD-N	1:1000 for WB	sc-393656	Santa Cruz (CA, USA)
GluR1	1:1000 for WB	05-855R	Millipore (MA, USA)
GluR2	1:1000 for WB	MABN71	Millipore (MA, USA)
NR1	1:1000 for WB	AB9864R	Millipore (MA, USA)
NR2A	1:1000 for WB	07-632	Millipore (MA, USA)
NR2B	1:1000 for WB	06-600	Millipore (MA, USA)
Synapsin 1	1:1000 for WB	MABN894	Millipore (MA, USA)
PSD95	1:1000 for WB	#3450	Cell Signaling Technology (MA, USA)
p-CREB	1:1000 for WB	05-807	Millipore(MA, USA)
CREB	1:1000 for WB	04-767	Millipore (MA, USA)
p-IRE1α	1:1000 for WB	ab48187	Abcam (Cambridge, UK)
IRE1α	1:1000 for WB	#3294	Cell Signaling Technology (MA, USA)
p-PERK	1:1000 for WB	DF7576	Affinity (Jiangsu, China)
PERK	1:1000 for WB	#3192	Cell Signaling Technology (MA, USA)
Cleaved-ATF-6	1:1000 for WB<br>1:100 for IHC	sc-166659	Santa Cruz (CA, USA)
ATF-6	1:1000 for WB	#65880	Cell Signaling Technology (MA, USA)
GRP78	1:1000 for WB	AF0729	Affinity (Jiangsu, China)
CHOP	1:1000 for WB<br>1:200 for IHC	#2895	Cell Signaling Technology (MA, USA)
XBP-1s	1:1000 for WB<br>1:200 for IHC	83418	Cell Signaling Technology (MA, USA)
p-NF-κB	1:1000 for WB	AB3375	Millipore (MA, USA)
NF-κB	1:1000 for WB	ABE347-25UG	Millipore (MA, USA)
p38	1:1000 for WB	#8690	Cell Signaling Technology (MA, USA)
p-p38	1:1000 for WB	#9211	Cell Signaling Technology (MA, USA)
Tubulin	1:1000 for WB	#2128	Cell Signaling Technology (MA, USA)
β-Actin	1:10000 for WB	#4967	Cell Signaling Technology (MA, USA)

Table S2. Primers used in PCR.

Primer	Sequence (5'-3')
mmu-miR-9-5p-RT	GTCGTATCCAGTGCGTGTCGTGGAGTCGGCAATTGCACTGGATAC GACTCATACAG
mmu-miR-9-5p-F	TCTTTGGTTATCTAGCTGT
mmu-miR-185-3p-RT	GTCGTATCCAGTGCGTGTCGTGGAGTCGGCAATTGCACTGGATAC GACACCAGAGG
mmu-miR-185-3p-F	AGGGGCTGGCTTTCCT
mmu-miR-7670-3p-RT	GTCGTATCCAGTGCGTGTCGTGGAGTCGGCAATTGCACTGGATAC GACTCTACAGA
mmu-miR-7670-3p-F	TTCCCTTTCTGAATATCT
mmu-miR-7676-3p-RT	GTCGTATCCAGTGCGTGTCGTGGAGTCGGCAATTGCACTGGATAC GACTGTGGGCA
mmu-miR-7676-3p-F	TCCGGTGCTCACTCTGC
mmu-miR-126a-3p-RT	GTCGTATCCAGTGCGTGTCGTGGAGTCGGCAATTGCACTGGATAC GACCGCATTAT
mmu-miR-126a-3p-F	TCGTACCGTGAGTAATA
mmu-miR-22-5p-RT	GTCGTATCCAGTGCGTGTCGTGGAGTCGGCAATTGCACTGGATAC GACTAAAGCTT
mmu-miR-22-5p-F	AGTTCTTCAGTGGCAAG
UR	CAGTGCGTGTCGTGGAGT
m-u6-f	CTCGCTTCGGCAGCACA
m-u6-r	AACGCTTCACGAATTTGCGT
M-Gapdh_F	GTGTTCCTACCCCCAATGTGT
M-Gapdh_R	ATTGTCATACCAGGAAATGAGCTT

Table S3. Sequences of synthetic miRNA mimics.

Primer	Sequence (5-3')
mmu-miR-7670-3p sence	UUCCCUUUCUGAAUAUCUGUAGA
mmu-miR-7670-3p anti-sence	UACAGAUAUUCAGAAAGGGAAUU
mimics-nc sense	UUCUCCGAACGUGU-CACGUTT
mimics-nc anti-sense	ACGUGACACGUUCGG-AGAATT

Table S4. Sequences of synthetic miRNA inhibitor.

Primer	Sequence (5-3’)
mmu-miR-7670-3p inhibitor	UCUACAGAUAUUCAGAAAGGGAA
inhibitor-nc	CAGUACUUUUGUGUAGUACAA

References

[1] L. Tao, Q. Liu, F. Zhang, Y. Fu, X. Zhu, X. Weng, H. Han, Y. Huang, Y. Suo, L. Chen, X. Gao, X. Wei, *Light, science & applications* **2021**, *10*, 179.
